# Selective arm-usage of pre-miR-1307 dysregulates angiogenesis and affects breast cancer aggressiveness

**DOI:** 10.1186/s12915-025-02133-x

**Published:** 2025-01-23

**Authors:** Oyku Ece Sumer, Korbinian Schelzig, Janine Jung, Xiaoya Li, Janina Moros, Luisa Schwarzmüller, Ezgi Sen, Sabine Karolus, Angelika Wörner, Verônica Rodrigues de Melo Costa, Nishanth Belugali Nataraj, Efstathios-Iason Vlachavas, Clarissa Gerhäuser, Karin Müller-Decker, Dominic Helm, Yosef Yarden, Birgitta Elisabeth Michels, Cindy Körner

**Affiliations:** 1 Division of Molecular Genome Analysis, German Cancer Research Center (DKFZ), Im Neuenheimer Feld 580, Heidelberg, 69120 Germany; 2 Faculty of Biosciences, University of Heidelberg, Im Neuenheimer Feld 234, Heidelberg, 69120 Germany; 3 Medical Faculty Heidelberg, University of Heidelberg, Im Neuenheimer Feld 672, Heidelberg, 69120 Germany; 4 MCBI program, Department of Biology, Faculty of Science, University of Tübingen, Tübingen, 72074 Germany; 5 Department of Biological Regulation, Weizmann Institute of Science, Rehovot, 76100 Israel; 6 Division of Cancer Epigenomics, German Cancer Research Center (DKFZ), Im Neuenheimer Feld 280, Heidelberg, 69120 Germany; 7 Tumor Models Core Facility, German Cancer Research Center (DKFZ), Im Neuenheimer Feld 280, Heidelberg, 69120 Germany; 8 Proteomics Core Facility, German Cancer Research Center (DKFZ), Im Neuenheimer Feld 280, Heidelberg, 69120 Germany

**Keywords:** Breast cancer, Angiogenesis, miRNAs, IsomiRs, miR-1307, miRNA arm switch, Selective arm-usage of miRNA, Metastasis

## Abstract

**Background:**

Breast cancer is the leading cause of cancer-related mortality in women. Deregulation of miRNAs is frequently observed in breast cancer and affects tumor biology. A pre-miRNA, such as pre-miR-1307, gives rise to several mature miRNA molecules with distinct functions. However, the impact of global deregulation of pre-miR-1307 and its individual mature miRNAs in breast cancer has not been investigated in breast cancer, yet.

**Results:**

Here, we found significant upregulation of three mature miRNA species derived from pre-miR-1307 in human breast cancer tissue. Surprisingly, the overexpression of pre-miR-1307 in breast cancer cell lines resulted in reduced xenograft growth and impaired angiogenesis*.* Mechanistically, overexpression of miR-1307-5p altered the secretome of breast cancer cells and reduced endothelial cell sprouting. Consistently, expression of miR-1307-5p was inversely correlated with endothelial cell fractions in human breast tumors pointing at an anti-angiogenic role of miR-1307-5p. Importantly, the arm usage of miR-1307 and other miRNAs was highly correlated, which suggests an undefined common regulatory mechanism.

**Conclusions:**

In summary, miR-1307-5p reduces angiogenesis in breast cancer, thereby antagonizing the oncogenic effects of miR-1307-3p. Our results emphasize the importance of future research on the regulation of miRNA arm selection in cancer. The underlying mechanisms might inspire new therapeutic strategies aimed at shifting the balance towards tumor-suppressive miRNA species.

**Graphical Abstract:**

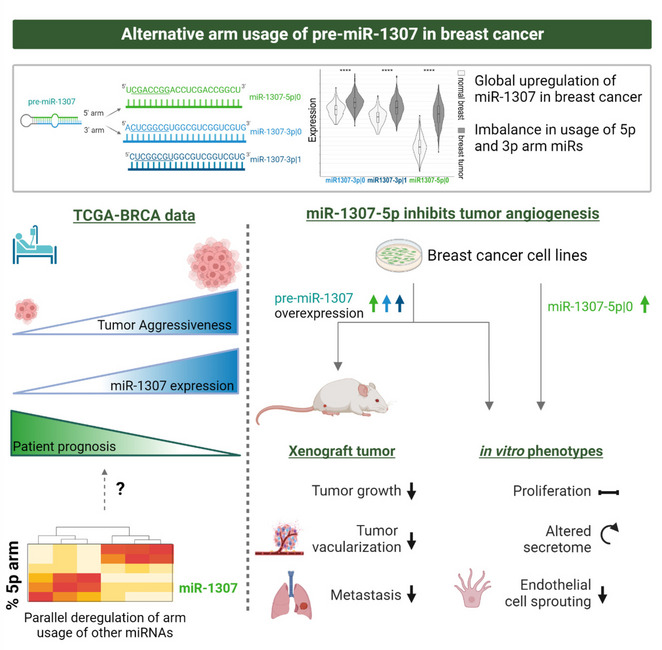

**Supplementary Information:**

The online version contains supplementary material available at 10.1186/s12915-025-02133-x.

## Background

Breast cancer remains the most prevalent form of malignancy and has the highest tumor-associated mortality in women worldwide [1]. Four intrinsic breast cancer subtypes (PAM50) were identified in the early 2000s based on gene expression profiles, allowing for improved risk stratification [2]. These PAM50 subtypes, which are still an important basis for clinical decisions today, include the two estrogen receptor-dependent subtypes, luminal A (LumA) and luminal B (LumB), the Human epidermal growth factor receptor 2 (HER2)-positive subtype and basal-like (basal) breast cancer. In particular, patients with LumA breast cancer have a favorable prognosis, whereas patients diagnosed with basal-like breast cancer face a higher risk of relapse, metastasis, and disease-associated mortality [2, 3].

MicroRNAs (miRs) are endogenous small non-coding RNAs and have a wide range of regulatory functions in normal and cancer cells [4]. They are transcribed by RNA polymerase II and cleaved by the microprocessor complex into a 70–80 nucleotide long, stem-looped precursor-miRNA (pre-miRNA) [5]. Pre-miRNAs are then transported to the cytoplasm and further processed into a 20–22 nucleotide long double-stranded miRNA duplex [6]. One of the strands is loaded into the RNA-induced silencing complex (RISC) and facilitates the downregulation of its specific targets by binding to the 3′UTR regions of target mRNAs [7]. The miRNA strand that is not loaded into the RISC is degraded [4, 8]. While most pre-miRNAs display a clear strand preference, a subset can produce mature miRNAs from both the 5′ (5p) and 3′ (3p) arms of their hairpin structure [9]. This balance can be shifted as an additional fine-tuning mechanism influenced by factors such as tissue type or developmental stage [9–11]. While the underlying molecular mechanisms governing miRNA arm selection are not fully understood, several studies have shown that miR-5p and miR-3p derived from the same pre-miRNA may have different regulatory functions and distinctive expression patterns in cancer [12–14].

Deep sequencing studies have uncovered that many pre-miRNAs can generate more than two mature miRNA variants, known as isomiRs, in addition to the canonical 5p and 3p arm variants [15, 16]. IsomiRs are length and/or sequence variants of the canonical mature miRNA; specifically, 5′isomiRs have a shifted 5′end resulting in altered seed sequences. Especially in cancer, several studies have shown that 5′isomiRs have distinct functional targets and phenotypic roles when compared to their canonical miRNAs [17–20].

Both the miRNA arm usage and 5′isomiRs constitute fundamental mechanisms of the functional evolution of pre-miRNAs and add to the complexity of the non-coding RNA regulatory network in cancer. Therefore, the combined analysis of these two features is pivotal to understanding the molecular mechanisms leading to malignant phenotypes.

In the present study, we investigated the role of pre-miR-1307 in breast cancer. This pre-miRNA gives rise to substantial levels of mature miRNAs from both arms, as well as to a highly expressed non-canonical 5′isomiR from the 3p-arm. Individual mature miRNAs derived from pre-miR-1307 have previously been associated with the development and progression of several cancer types [21–24]. However, the impact of pre-miR-1307 as a whole on breast cancer progression has not yet been characterized.

Therefore, we specifically addressed the expression levels and functions of 5′isomiRs derived from pre-miR-1307, namely miR-1307-3p|0, −3p|1, and −5p|0. We found that all three expressed mature 5′isomiRs were overexpressed in breast cancer, especially in the basal-like subtype. Surprisingly, the overexpression of pre-miR-1307 in human basal-like breast cancer cells inhibited metastasis and angiogenesis in vivo. We show that miR-1307-5p mediates this tumor suppressive phenotype and that its expression is associated with increased hypoxic conditions, as well as reduced expression of angiogenesis-related genes in patients’ tumor samples. Importantly, we found a substantial correlation of arm usage between pre-miR-1307 and 30 other pre-miRNAs in breast cancer, suggesting a coordinated regulatory mechanism of these miRNAs with potential further phenotypic implications.

## Results

### Mature miRNAs derived from pre-miR-1307 are commonly overexpressed in breast tumor samples

**Fig. 1:**
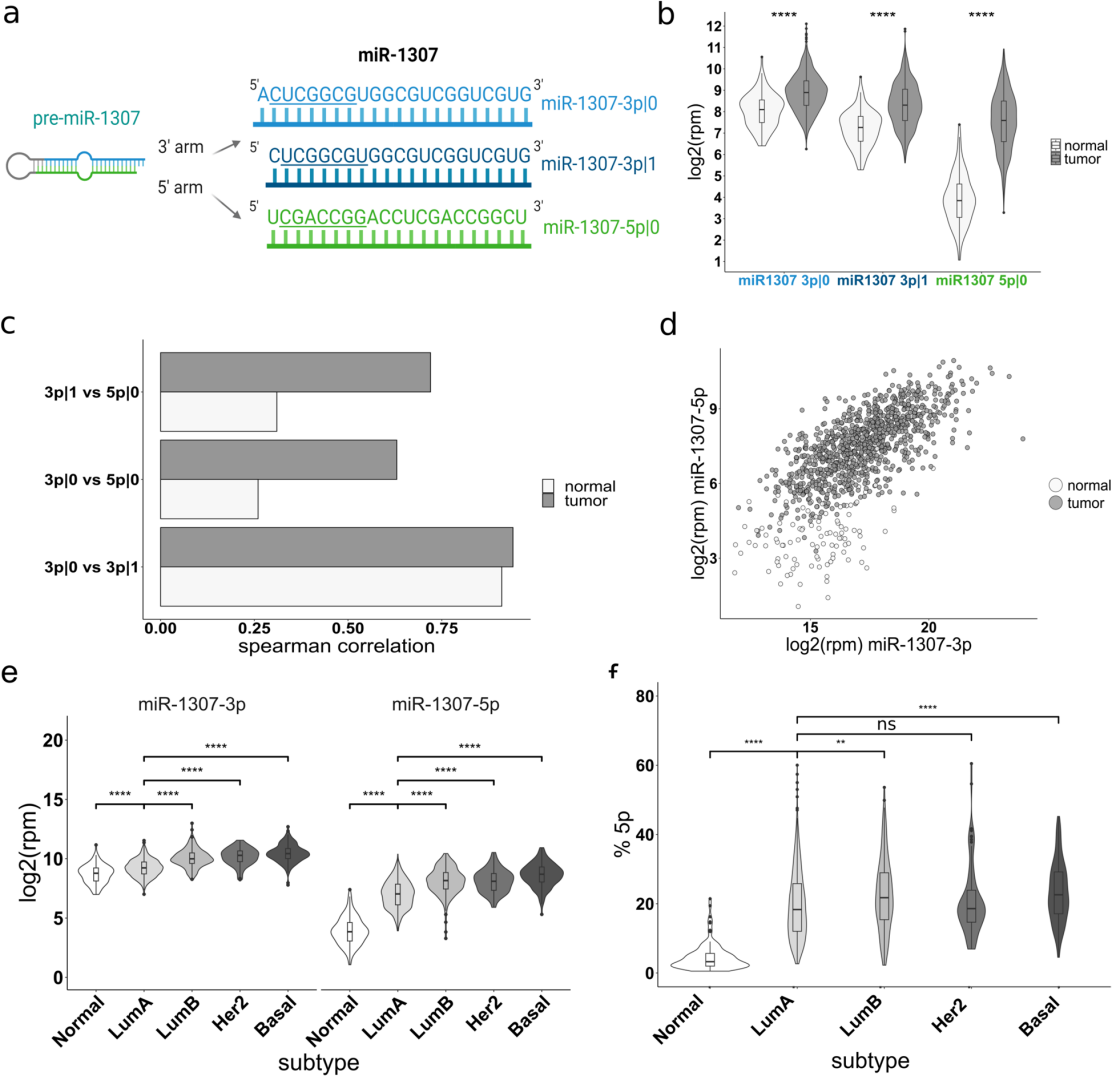
Mature miRNAs derived from pre-miR-1307 are commonly overexpressed in TCGA breast tumor samples. **a** Mature 5′isomiRs expressed from the pre-miR-1307 locus. Seed sequences are underlined. **b** Batch-corrected isomiR expression data of breast cancer patients from the TCGA-BRCA cohort were obtained from GSE164767 and collapsed to 5′isomiR expression. Log2 reads per million (rpm) values were plotted for the individual 5′isomiRs of pre-miR-1307 for tumor samples and tumor-adjacent normal samples (referred to as “normal”). **c** The pairwise Spearman correlation between the three individual 5′isomiRs of pre-miR-1307 was calculated separately for tumor and tumor-adjacent normal tissue. Here, the bars next to the label “3p|1 vs 5p|0” indicate the Spearman correlation or miR-1307-3p|1 and miR-1307-5p|0 overall tumor (dark gray) or normal (light gray) samples in the dataset. **d** The expression of both arms of pre-miR-1307 was plotted across all patients. Tumor samples are colored in dark gray and tumor-adjacent normal tissue samples are colored in white. **e** The expression levels of miR-1307-3p and −5p were plotted for each PAM50 subtype and tumor-adjacent normal breast tissue. **f** The relative expression of miR-1307-5p in percent of all reads derived from mature miRNAs of pre-miR-1307 was calculated for tumor samples of the different PAM50 subtypes and tumor-adjacent normal tissue. **b**, **e**, **f** Statistical analysis was performed using an unpaired, two-tailed Student’s *t*-test. * *p* < 0.05, ** *p* < 0.01, *** *p* < 0.001, **** *p* < 0.0001

The expression of various miRNAs has been linked to tumor-associated phenotypes. One such example is miR-1307-3p|0, the canonical 5′isomiR derived from the 3p arm of pre-miR-1307, which has been described to promote cancer progression in certain cancer types [22, 23, 25]. However, most previous studies have focused on the role of the canonical miRNA expressed from the 3p arm without considering the role of the 5p arm or potential 5'isomiRs of miR-1307-3p|0. Hence, to investigate the potential relevance of other mature miRNAs originating from pre-miR-1307, we first characterized their expression patterns in breast cancer. To this end, we compared the expression levels of these 5′isomiRs between breast tumor tissue and tumor-adjacent normal breast tissue from the TCGA BRCA dataset. Of note, we summed the expression of all 3′isomiRs with the same 5′starting position to focus specifically on the variants with different seed sequences which are likely to result in different phenotypes. Here, we identified three different substantially expressed 5′isomiRs: miR-1307-3p|0, miR-1307-3p|1, both of which are derived from the 3p arm, and miR1307-5p|0, which originates from the 5p arm of the pre-miR (Fig. 1a). All three forms were present with various 3′isomiRs expressed at different levels (Additional File 1: Fig. S1). In the following, we will focus on the expression of the 5′isomiRs irrespective of their specific 3′end.

Of note, miR-1307-3p|1 is a 5′isomiR with a shifted seed sequence, which was expressed at similar levels as the canonical miR-1307-3p|0. The expression of all three mature isomiRs was significantly increased in the tumor samples with the most pronounced increase observed for miR-1307-5p|0 (Fig. 1b).

Because all three isomiRs are derived from the same pre-miRNA and therefore transcriptionally co-regulated, we anticipated a high correlation in their expression. To confirm this, we next systematically assessed the pairwise correlations between the isomiRs. As expected, the expression of miR-1307-3p|0 and −3p|1 was highly correlated both in cancerous and normal samples (Fig. 1c). In contrast, the correlation of miR-1307-5p|0 with both isomiRs from the 3p arm was less pronounced, especially in tumor-adjacent normal tissue. Due to the nearly perfect positive correlation of the 5′isomiRs from the 3p arm, subsequent analyses in patient samples focused on the comparison between the 5p and the 3p arm without accounting for 5′isomiRs (correlation visualized in Fig. 1d). The lower correlation of the miRNA arms of pre-miR-1307 in patient samples suggests a potential regulatory fine-tuning of their expression at the post-transcriptional level, especially when comparing tumor and normal samples.

Given the known molecular differences among PAM50 cancer subtypes, we next investigated if the upregulation of both arms was consistent across all subtypes. Here, the expression of both the 3p and the 5p arm of pre-miR-1307 was lower in normal breast tissue in comparison to LumA tumors, and further elevated in the more aggressive PAM50 subtypes. This suggests transcriptional upregulation irrespective of the breast cancer subtype, with the strongest effect in aggressive tumors (Fig. 1e). Of note, the expression differences for miR-1307-5p were more pronounced than for miR-1307-3p. To quantify this, we calculated the percentage of miRNA reads from miR-1307-5p|0 relative to all reads derived from pre-miR-1307 for each patient (Fig. 1f). Intriguingly, we observed a pattern similar to that of individual arm expression levels: the percentage was lower in tumor-adjacent normal tissue compared to LumA tumors and further elevated especially in aggressive basal-like and LumB tumors. This finding suggests that more aggressive tumor subtypes exhibit a more strongly de-regulated arm usage pattern compared to less aggressive LumA tumors.

In summary, all 5′isomiRs derived from pre-miR-1307 are overexpressed in breast cancer, but the extent of overexpression differs between the mature miRNAs derived from both arms. An increased correlation between the 3p forms and miR-1307-5p|0 together with the strongly increased expression level of the latter isoform suggest a shift of the arm preferences towards miR-1307-5p|0 in cancer tissue when compared to normal breast tissue. Together, this indicates distinct regulation and potentially distinct functions of the two arms of pre-miR-1307.

### Overexpression of pre-miR-1307 attenuates tumor growth of basal-like breast cancer cells in vivo without affecting cell proliferation in vitro

**Fig. 2:**
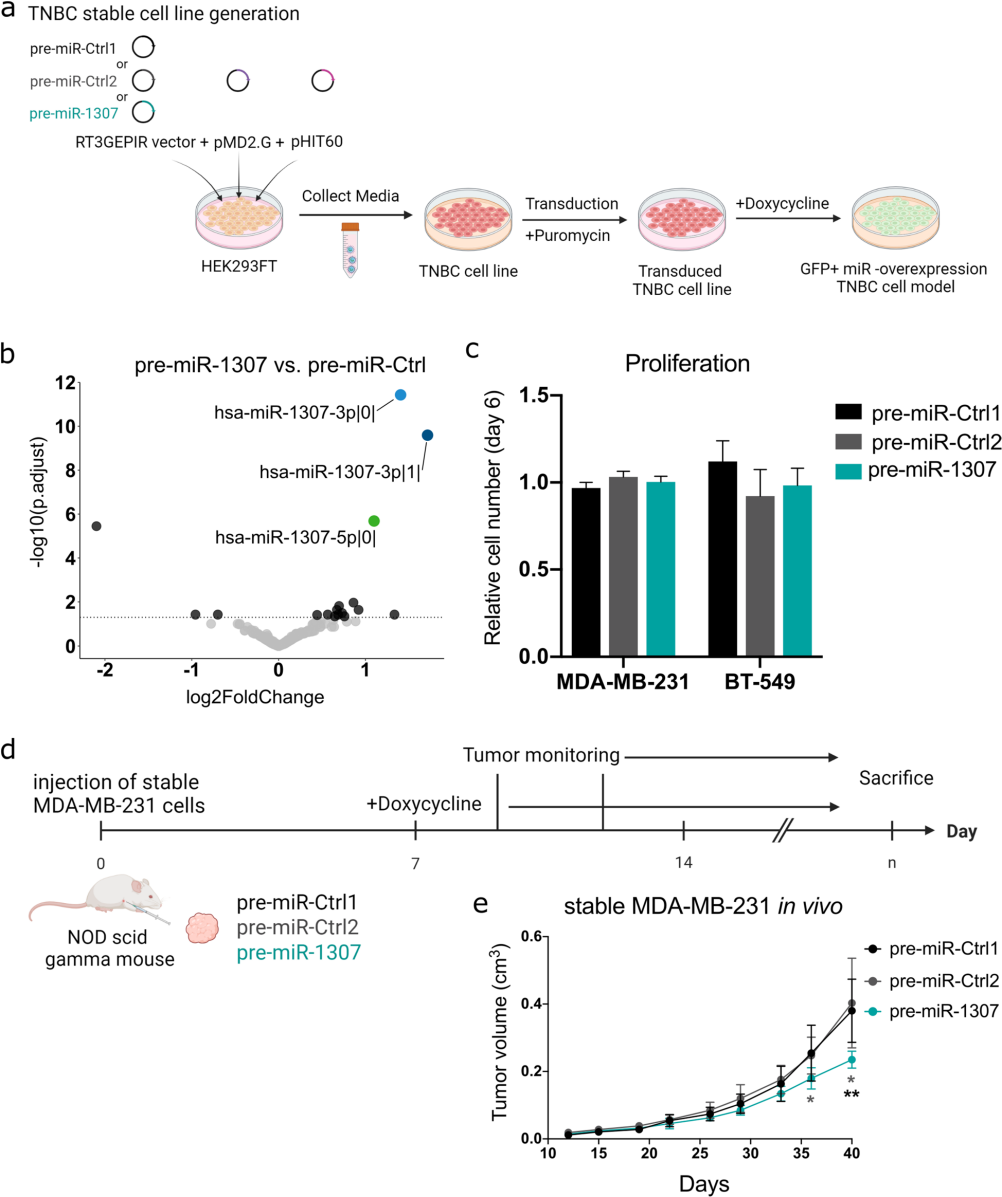
Overexpression of pre-miR-1307 attenuates tumor growth in vivo without affecting cell proliferation in vitro. **a** Basal breast cancer cell lines were stably transduced with doxycycline-inducible overexpression vectors to express pre-miR-1307 or two non-human control pre-miRNAs together with GFP. Transduced cells were positively selected with puromycin. **b** Overexpression of pre-miR-1307 in stably transduced MDA-MB-231 cells after 48 h induction with doxycycline was verified by small RNA sequencing in three replicates. A differential expression analysis between the 5′isomiR expression of cells overexpressing the non-human control pre-miRNAs and pre-miR-1307 was performed using DESeq2 and apeglm for shrinkage estimation. Significantly differentially expressed 5′isomiRs (*p* < 0.05) are highlighted in black. The 5′isomiRs derived from pre-miR-1307 are labeled. **c** MDA-MB-231 and BT-549 cells overexpressing pre-miR-1307 or two non-human pre-miRNAs were seeded in 96-well plates and pre-miRNA overexpression was induced. Cell numbers were assessed every day using Hoechst staining and fluorescence-based cell counting in a screening microscope. The change in cell number between day 1 and day 6 was normalized to the average value of the control pre-miRNAs. The mean and standard deviation of 3 independent replicates are shown. **d** Schematic representation of the xenograft mouse tumor growth experiment. **e** MDA-MB-231 cells with stable overexpression of pre-miR-1307 or two non-human control pre-miRNAs were orthotopically injected into the mammary fat pad of NOD Scid gamma mice (*n* = 5–6 per group). At day seven post-injection, pre-miRNA expression was induced by doxycycline. Tumor growth was measured twice a week and tumor volume was calculated. The mean and standard deviation of 5 or 6 replicate tumors are shown. Values obtained for pre-miR-1307 were compared to the pre-miRNA controls by unpaired, two-tailed Student’s *t*-test. * *p* < 0.05, **p < 0.01

To comprehensively characterize the phenotypic consequences of the overexpression of pre-miR-1307 in basal-like breast cancer cells, we next generated breast cancer cell lines (MDA-MB-231 and BT-549) with inducible expression of pre-miR-1307 or two *C.elegans* pre-miRNAs as negative controls (Fig. 2a). To validate that this system can be utilized to study the joint overexpression of the three substantially expressed 5′isomiRs discovered in the breast cancer patient data from TCGA, we performed miRNA sequencing to quantify 5′isomiR levels in MDA-MB-231 cells transduced with the different constructs. A differential expression analysis between pre-miR-1307 overexpressing cells and control cells revealed a mild, but consistent and highly significant upregulation of all three detected 5′isomiRs of pre-miR-1307, whereas no global changes in 5′isomiR expression could be observed (Fig. 2b). The overexpression levels of both miRNA arms were similar in our model, allowing us to study the phenotypic impact of the joint transcriptional upregulation of all miRNA species derived from pre-miR-1307.

Hence, we utilized this model to study the phenotypic impact of pre-miR-1307 overexpression in basal breast cancer cell lines. Since miR-1307-3p has previously been described to enhance the proliferation of cancer cells [25], we expected to observe enhanced proliferation upon overexpression of pre-miR-1307. However, contrary to our expectations, the overexpression of pre-miR-1307 did not affect proliferation in both breast cancer cell lines (MDA-MB-231 and BT549) tested (Fig. 2c). We therefore hypothesized that the tumor-promoting effect was not effective in our in vitro system and went on to investigate the impact of pre-miR-1307 overexpression on tumor progression in vivo. Of note, the investigation of individual mature miRNAs in xenograft models is hampered by the fact that there are no stable expression systems available. Therefore, in vivo analyses were limited to the overexpression of the complete hairpin, giving rise to all three mature isomiRs. To this end, we orthotopically injected MDA-MB-231 cells stably transduced with pre-miR-1307 or two different control miRNAs into the mammary fat pad of NOD scid gamma mice (*n* = 6 per control group and *n* = 5 for pre-miR-1307 overexpression). At day seven post-injection, when tumors were just palpable, miRNA expression was induced and tumor growth was monitored until a defined humane endpoint was reached (scheme see Fig. 2d). Successful induction of transgene expression within the size-matched tumors after the termination of the experiment was demonstrated by GFP expression in IHC sections (Additional File 1: Fig. S2). Tumor growth was attenuated when the injected tumor cells overexpressed pre-miR-1307 compared to cells harboring the control constructs (Fig. 2e). When the first mice had to be sacrificed (34 days after pre-miRNA induction), the average tumor volume of the control tumors were 0.38 cm^3^ and 0.37 cm^3^, respectively, whereas the average volume of pre-miR-1307 overexpressing tumors was 0.24 cm^3^ (*p* = 0.01, two-tailed unpaired *t*-test). Hence, tumor growth was reduced by 37%.

Mice were sacrificed once the humane endpoint was reached. Staining for proliferative cells using an antibody targeting Ki67 revealed that there was no significant difference in the proliferative capacity of the tumor cells at this time point (Additional File 1: Fig. S3).

In summary, mild upregulation of miR-1307 did not affect cell proliferation of MDA-MB-231 and BT-549 breast cancer cells in vitro or in vivo, however, significantly reduced tumor growth in vivo.

### Overexpression of pre-miR-1307 reduces metastasis

To further characterize the phenotypic effect of pre-miR-1307, we next investigated the potential of the tumors derived from the different stable cell lines to induce micrometastases in the lungs of the mice.

**Fig. 3:**
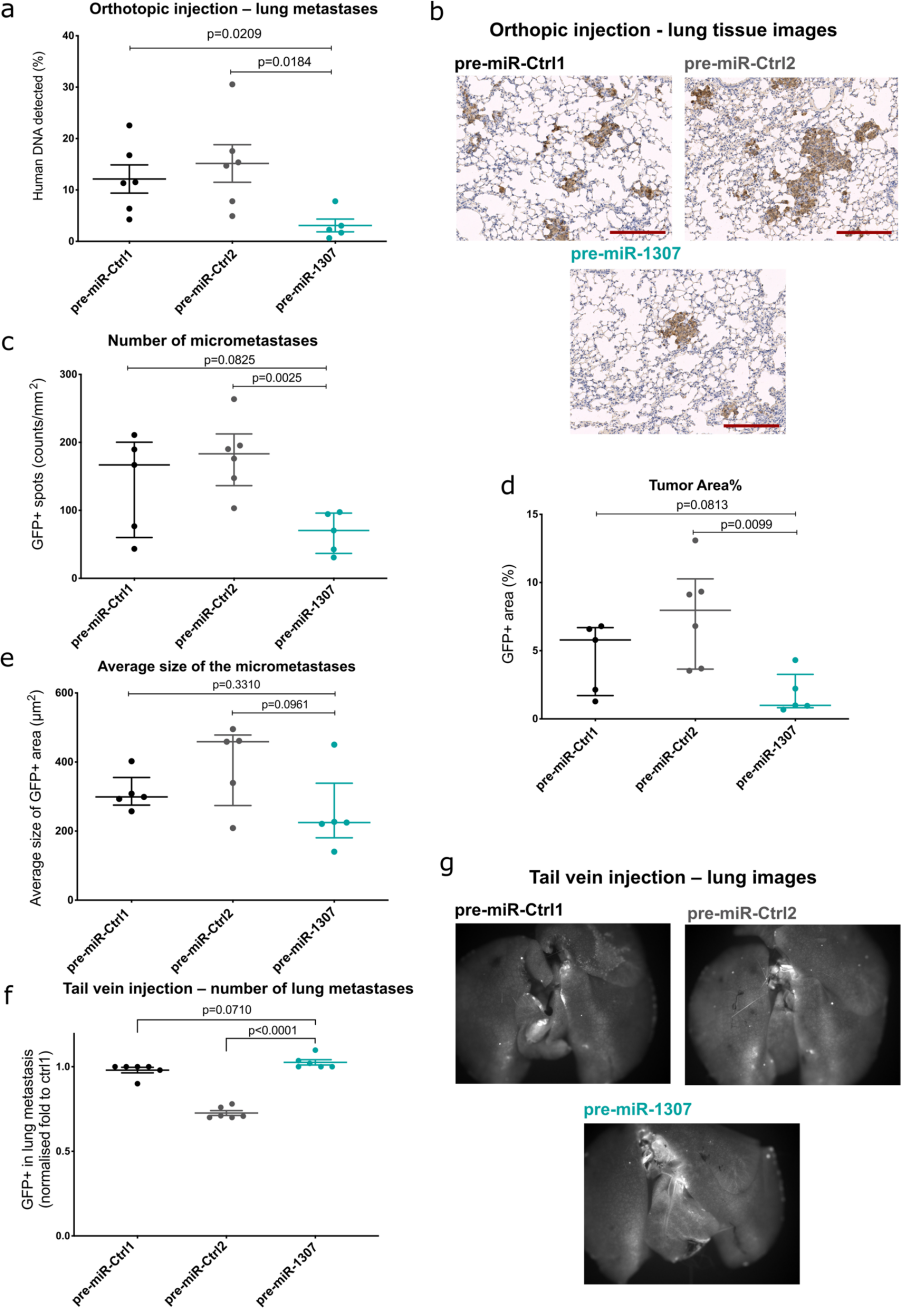
Overexpression of pre-miR-1307 inhibits metastasis in mouse tumors. **a**–**e** MDA-MB-231 cells with stable overexpression of pre-miR-1307 or two non-human control pre-miRNAs were orthotopically injected into the mammary fat pad of NOD Scid gamma mice (*n* = 5–6 per group; see Fig. 2, same mouse cohorts). At day seven post-injection, pre-miRNA expression was induced by administration of doxycycline in drinking water. Once one of the humane endpoints was reached for a mouse, it was sacrificed and micrometastases in the lungs were investigated. **a** The relative content of human DNA in the murine lungs was quantified by qPCR with primers targeting human *Alu* sequences. Obtained C_t_ values were transformed to the percentage of human DNA in the sample by comparison with a standard curve (Additional File 1: Fig. S4). **b**–**e** FFPE tissue sections of the lungs were used for IHC staining using an anti-GFP antibody to detect pre-miRNA overexpressing cells as an indicator of micrometastases. The stainings were quantified using ImageJ macros provided by the Imaging Core Facility of DKFZ **b** Representative stained tissue sections. Scale bar: 200 μm. **c** The number of GFP-positive spots above a size of 170 pixels was counted. (d) The relative GFP-positive area per lung section was quantified based on the IHC staining. **e** The average size of the GFP-positive areas was assessed for each lung section. **c**–**e** Each dot represents the value obtained from one mouse (*n* = 5–6 mice per group). Data are presented as the median ± IQR (interquartile range). Statistical comparisons were performed using an unpaired, two-tailed Student’s *t*-test. **f** and **g** MDA-MB-231 cells stably transduced with pre-miR-1307 or one of two non-human control pre-miRNAs were induced to overexpress the pre-miRNAs together with GFP using doxycycline for 72 h in vitro. Next, cells were injected into the tail vein of NOG mice (*n* = 6 per group) and mice were sacrificed 3 weeks later. Lungs were excised, photographed, and quantified metastatic nodules based on the GFP signal. **f** The data are presented as averages ± SEM. **g** Shown are representative fluorescence images of the lungs of each group

To this end, we sacrificed each mouse once the respective tumor reached the predefined maximal size of 1 cm, harvested the lungs, and quantified the relative abundance of human cells in the murine lungs by *Alu*-PCR. Here, we observed a significant reduction in the relative proportion of human DNA in the lungs of mice bearing pre-miR-1307 overexpressing tumors compared to the controls (size-matched tumors, Fig. 3a). IHC analysis of lung sections confirmed these findings as both the number of GFP + foci in the lungs and consequently of the relative GFP + areas showed the trend to be reduced under pre-miR-1307 overexpression conditions (Fig. 3B–D). Of note, the average size of individual GFP + micrometastases did not differ between the different conditions (Fig. 3E).

We hypothesized that the reduced number of micrometastases was caused by impaired colonization capacities of tumor cells overexpressing pre-miR-1307. To test this, we next performed tail vein injection of the stably transduced MDA-MB-231 cells using less severely immunocompromised NOG mice. Ex vivo fluorescence imaging of the GFP co-expressed with the pre-miRNAs showed that the number of metastatic signals was not reduced by the overexpression of pre-miR-1307. While there was no significant difference compared to pre-miR-Ctrl1, pre-miR-1307 even induced metastasis compared to pre-miR-Ctrl2, suggesting that extravasation and colonization were not impaired by miR-1307 (Fig. 3F,G).

Taken together, our results imply that pre-miR-1307 interferes with tumor growth and metastatic dissemination in vivo by a mechanism independent from cell proliferation and from their potential to colonize distant organs.

### Overexpression of pre-miR-1307 impairs tumor angiogenesis

**Fig. 4:**
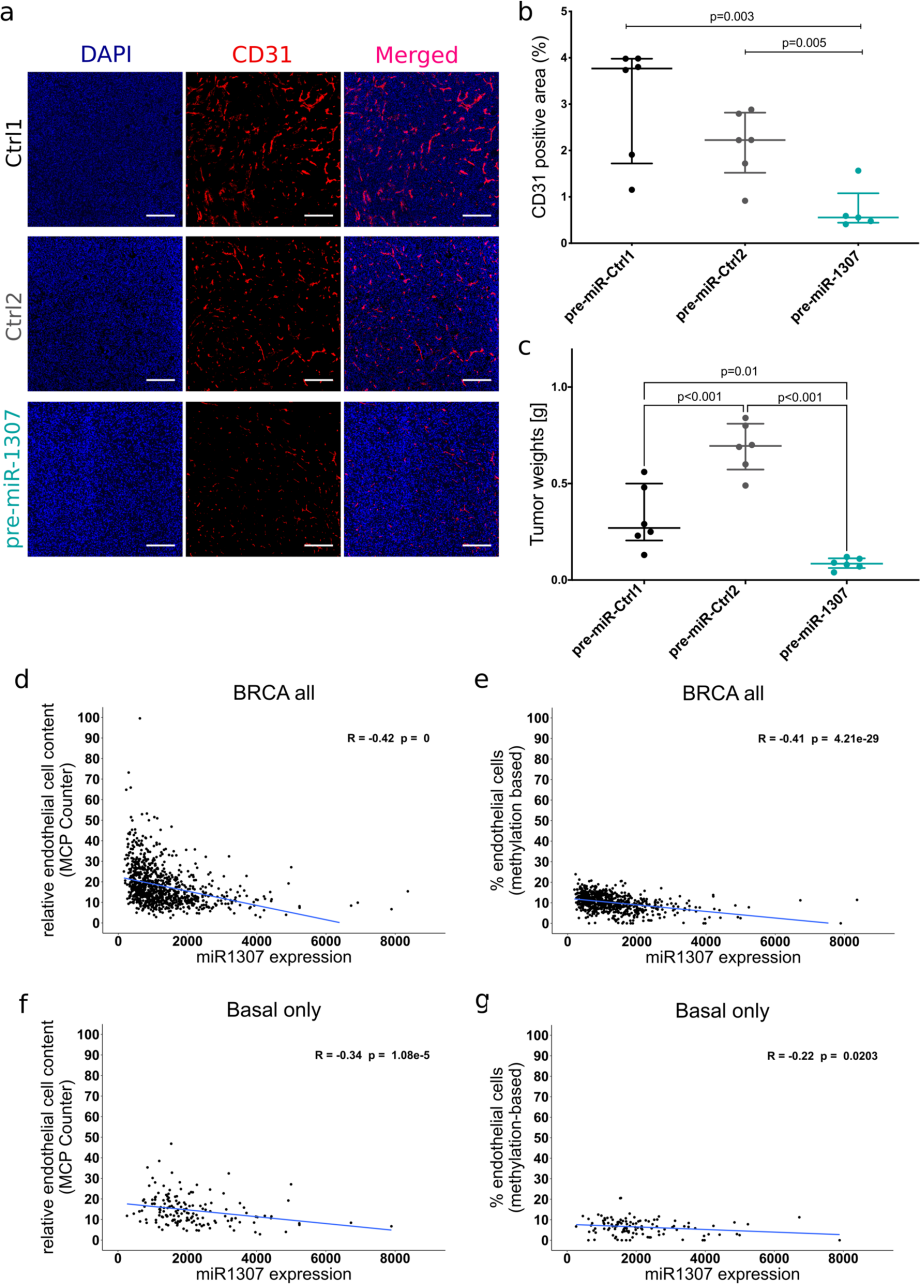
Overexpression of pre-miR-1307 impairs tumor angiogenesis in mouse tumors. **a**–**b** MDA-MB-231 cells with stable overexpression of pre-miR-1307 or two non-human control pre-miRNAs were orthotopically injected into the mammary fat pad of NOD Scid gamma mice (*n* = 5–6 per group). Seven days after injection, pre-miRNA expression was induced by administration of doxycycline in drinking water. Once one of the humane endpoints was reached for a mouse, it was sacrificed and CD31 expression in the primary tumors was investigated by immunofluorescence analysis on cryosections. DNA was counterstained with Hoechst. **a** Exemplary images of CD31 staining (scale bar: 100 µm) and **b** quantification of the CD31-positive area per tissue sample. **c** MDA-MB-231 cells with stable overexpression of pre-miR-1307 or two non-human control pre-miRNAs were orthotopically injected into the mammary fat pad of NOD Scid gamma mice (*n* = 5–6 per group). Pre-miRNA expression was induced by the administration of doxycycline in drinking water directly after injection and 69 days after injection, mice were sacrificed and tumor weights were measured. **b**, **c** Statistical analysis was performed as an unpaired, two-tailed Student’s *t*-test. **d**–**g** The overall expression of miR-1307, which is defined as the sum of miR-1307-3p and −5p per sample of the TCGA-BRCA cohort, was determined and correlated with the endothelial cell content determined by **d**, **f** the RNA-Seq-based method MCP Counter, or by **e**, **g** a methylation-based approach for **d**–**e** all BRCA tumors and **f**–**g** PAM50 basal tumors. Spearman correlation coefficients, *p*-values, and linear trendlines are displayed in the scatterplots

Since angiogenesis and tumor vascularization are required for tumor outgrowth but not for initial metastatic colonization, we hypothesized that pre-miR-1307 might impair vascularization. To test this hypothesis, we performed immunofluorescence staining for CD31, a marker for endothelial cells and blood vessels, in sections of size-matched primary xenograft tumors derived from the first xenograft experiment. Quantitative analysis of the CD31-positive area revealed significantly reduced vascularization in pre-miR-1307 overexpressing tumors compared to both controls, supporting an inhibitory function of pre-miR-1307 on tumor angiogenesis (Fig. 4a,b).

To corroborate our findings, we conducted a second xenograft experiment with pre-miR-1307 overexpression by administration of doxycycline in drinking water directly after orthotopic injection of the cells. In line with our hypothesis that expression of pre-miR-1307 would prevent angiogenesis and, thereby, also the formation of large tumors, pre-miR-1307 overexpressing MDA-MB-231 cells failed to grow palpable tumors in less severely immunocompromised NOG mice, while control cells formed tumors also in this model. Resection of the mammary glands revealed the presence of small tumors with significantly reduced size compared to the controls (Fig. 4c).

To validate the relevance of this observation in the context of human breast tumor tissue, we assessed the association between the expression of miR-1307 and the presence of endothelial cells in the tumor samples subjected to molecular profiling within the TCGA project. For that purpose, we employed MCP Counter and methylation-based cell type deconvolution to estimate the relative endothelial cell content. These estimates were then correlated with the expression of pre-miR-1307 determined as the sum of expression of the three isomiRs using either all tumor samples (Fig. 4d,e) or tumor samples with a PAM50 subtype “basal” (Fig. 4f,g). Here, we observed significant inverse correlations between pre-miR-1307 expression and endothelial cell content estimated by both independent methods across all breast tumor samples (spearman correlation, MCPCounter *r*_*s*_ = − 0.42, *p* < 0.001; methylation-based estimation *r*_*s*_ = − 0.41, *p* < 0.05). The inverse correlations were weaker but still significant also within the subset of patients with PAM50 subtype “basal,” which reflects the subtype of our in vitro model system (Spearman correlation, MCPCounter *r*_*s*_ = − 0.34, *p* < 0.001; methylation-based estimation *r*_*s*_ = − 0.20, *p* < 0.05).

Taken together, these results demonstrate — both in our in vivo xenograft model employing ectopic overexpression of pre-miR-1307 and in human breast tumor tissue — an inverse correlation between the abundance of pre-miR-1307 and the presence of CD31-positive endothelial cells. Since the vascularization in tumor tissue is largely dependent on the induction of angiogenesis initiated by endothelial cell sprouting, these observations might suggest a functional implication of pre-miR-1307 in this process.

### miR-1307-5p expression in breast cancer cells inhibits sprouting capacity of endothelial cells via altered protein secretion

**Fig. 5:**
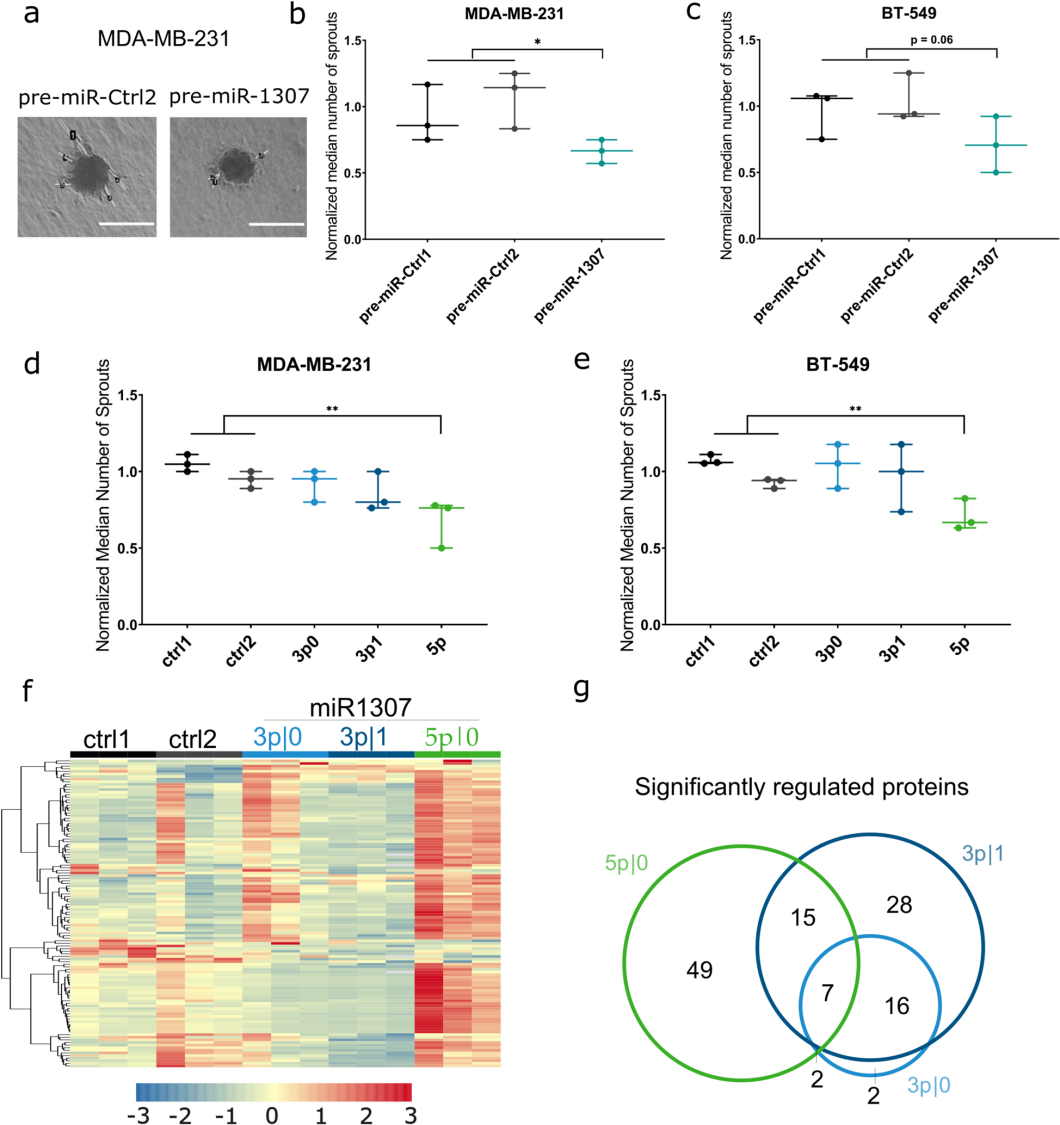
miR-1307-5p expression in breast cancer cells inhibits sprouting of endothelial cells via altered protein secretion. **a**–**e** HUVEC endothelial cells were grown as spheres and allowed to sprout for 24 h in the presence of conditioned media of breast cancer cells. **a** Representative images of sprouts generated in response to incubation with conditioned media of stably transduced MDA-MB-231 cells (scale bar: 100 µm). The median number of sprouts per sphere of at least eight spheres per condition was determined using conditioned media of stably transduced **b** MDA-MB-231 and **c** BT-549 cells or of transiently transfected **d** MDA-MB-231 and **e** BT-549 cells and normalized to the average of both controls from the respective replicate (*n* = 3 independent experiments). The number of sprouts was compared between conditioned media from cells **b**–**c** overexpressing pre-miRNA-1307 and both controls or **d**–**e** transfected with mimics of the 5′isomiRs derived from pre-miRNA-1307 and both controls using a two-tailed unpaired Student’s *t*-test. *p < 0.05, **p < 0.01. **f**–**g** Conditioned media obtained from MDA-MB-231 cells transfected with mimics of the 5′isomiRs derived from pre-miRNA-1307 and both controls was collected and subjected to mass spectrometry-based secretome analysis (*n* = 3 replicates per condition). The abundance of all proteins in the miRNA overexpression conditions was compared to the controls by unpaired Student’s *t*-test and *p*-values were corrected for multiple testing (Benjamini-Hochberg). **f** *Z*-scaled abundance of proteins with significant differences between conditions visualized as a heatmap. **g** The proteins with a significant difference (BH-adjusted *p*-value < 0.05) between at least one miRNA overexpression sample and the controls were displayed in a Venn diagram showing which miRNA mimic caused a significant change

To establish a functional link between the expression of pre-miR-1307 in cancer cells and their potential to secrete factors that induce the sprouting of endothelial cells, we next performed an in vitro sprouting assay. For that purpose, we treated spheroids of endothelial cells with conditioned media, that contains the secretomes of MDA-MB-231 or BT-549 cells stably overexpressing pre-miR-1307 or control pre-miRNAs. Indeed, we observed a reduction in the ability of the conditioned media from cells overexpressing pre-miR-1307 to induce endothelial cell sprouting as indicated by a consistent reduction in the number of sprouts per spheroid (Fig. 5a–c). This change was statistically significant in media derived from MDA-MB-231 cells (*p* < 0.05), and there was a trend towards significance in media derived from BT-549 cells (*p* = 0.06).

Next, we aimed to dissect the role of the individual mature isomiRs derived from pre-miR-1307 in vitro. For that purpose, we repeated the HUVEC endothelial cell sprouting assays using conditioned media of basal-like breast cancer cells transfected with miRNA mimics representing the individual isomiRs (Fig. 5e,f). We observed a mild but significant decrease in sprouting capacity, when the endothelial cells were incubated with conditioned media from breast cancer cells overexpressing miR-1307-5p|0. This was true for both MDA-MB-231 and BT-549 cells. In contrast, neither of the 5′isomiRs derived from the 3p-arm of pre-miR-1307 affected the sprouting. These data indicate that the expression of specifically miR-1307-5p|0 in the breast cancer cells results in changes in their secretome, which impairs the ability of the breast cancer cells to induce endothelial cell sprouting as a proxy for angiogenesis.

This finding was further corroborated by mass spectrometry-based analysis of the secretomes of isomiR-mimics-transfected MDA-MB-231 cells (Fig. 5f, Additional File 2: Table S1): While the proteins detected in the secretomes of cells overexpressing miR-1307-3p|0 and |1 were similar to both controls, secretomes of cells transfected with miR-1307-5p|0 were clearly distinct. Notably, most differentially detected proteins were increased in the conditioned media in response to miR-1307-5p|0 overexpression (Fig. 5f).

When comparing proteins significantly regulated by the different mature forms of pre-miR-1307, we observed overlapping but also distinct proteins regulated by the 5′isomiRs derived from the 3p-arm. Notably, the overexpression of miR-1307-5p led to the deregulation of a distinct set of secreted proteins when compared to the 3p forms (Fig. 5g). For instance, the well-known anti-angiogenic Thrombospondin-1 (encoded by the *THBS1* gene) was specifically upregulated in conditioned media of miR-1307-5p overexpressing cells, making it a potential mediator of the anti-angiogenic phenotype. However, the coordinated de-regulation of diverse factors with pro- and anti-angiogenic features suggests that the effect is multi-factorial and not confined to the regulation of a single key protein (see Additional File 2: Table S1 for the complete list of regulated proteins).

In summary, miR-1307-5p exhibited an effect of protein secretion, which was distinct from the effect of both isomiRs derived from miR-1307-3p. Phenotypically, these changes were reflected in a reduced potential of the respective conditioned media to induce sprouting of endothelial cells. This suggests a tumor suppressive role of miR-1307-5p in breast cancer induced by the coordinated de-regulation of a plethora of secreted factors.

### Arm usage of pre-miR-1307 is associated with breast cancer prognosis and with arm usage of other pre-miRNAs

Next, we aimed to further investigate the potential tumor suppressive function of miR-1307-5p via impairing tumor angiogenesis in breast tumor tissue. For that purpose, we again analyzed the data obtained from TCGA, but this time with an arm-level resolution.

**Fig. 6:**
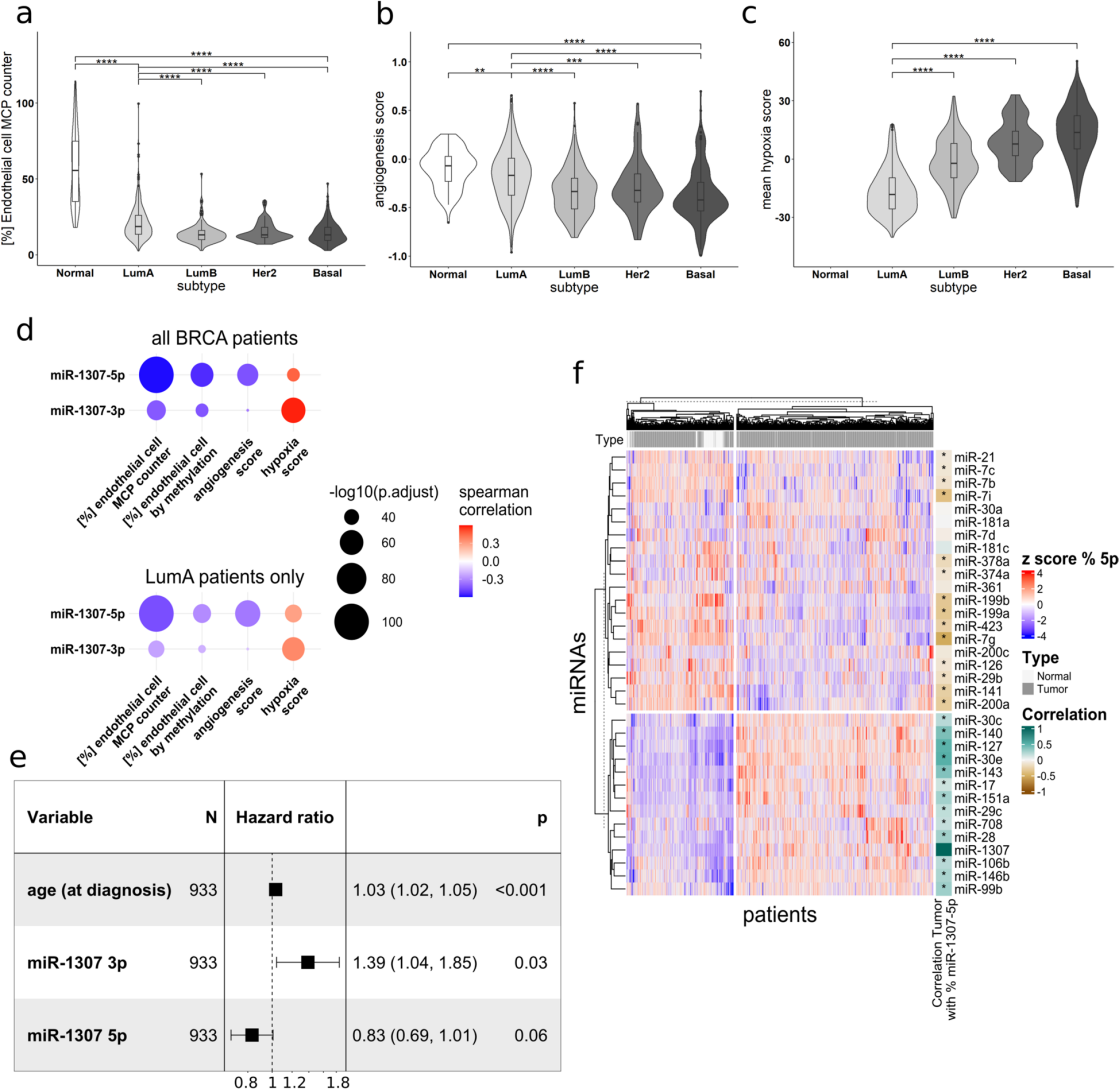
Arm usage of pre-miR-1307 is associated with patient prognosis and arm usage of other pre-miRNAs. Data was obtained from the TCGA BRCA project. **a** The endothelial cell content for each sample was estimated based on mRNA expression using MCP Counter and plotted for tumor-adjacent normal tissue (referred to as “normal” or the PAM50 subtypes of breast cancer. **b** The relative mRNA expression of the genes within the Hallmark gene set “Angiogenesis” (MSigDB) was used to determine an “angiogenesis score” for each sample. A high score indicates a high expression of these angiogenesis-related genes. **c** The Hypoxia Scores (Winter, Ragnum, and Buffa) were downloaded from cBioPortal. The mean of the scores was calculated for each patient and plotted for the different PAM50 subtypes of breast cancer. No Hypoxia Scores could be obtained for tumor-adjacent normal samples. **a**–**c** Statistical analysis was performed as an unpaired, two-tailed Student’s *t*-test. * *p* < 0.05, ** *p* < 0.01, *** *p* < 0.001, **** *p* < 0.0001. **d**–**g** Batch-corrected isomiR expression values were obtained from GSE164767 and collapsed to 5′isomiR expression and miRNA arm-level expression. The Spearman correlation coefficients and p-values for the correlations between miR-1307-5p|0, −3p|0, and 3p|1 and the endothelial cell content estimated using MCP Counter or DNA methylation, the mean Hypoxia Score and the Angiogenesis Score were calculated **d** across all breast cancer samples and across breast cancer samples of the PAM50 subtypes “luminal A.” **e** Clinical information about the age of the patients and overall survival was obtained from the TCGA project. A Cox PH regression analysis was performed using the PAM50 subtypes as strata and age at diagnosis, miR-1307-3p and miR-1307-5p expression as factors. **f** The *z*-scaled relative expression of all miRNAs expressed from the 5p arm was subjected to unsupervised clustering (Euclidian distance, WardD). The Spearman correlation with the relative expression of miR-1307-5p across all samples was annotated and marked with * for *p* < 0.05

First, we assessed the association between the isomiR levels and the endothelial cell content, activity of an angiogenesis gene signature, and hypoxia within the tumors. Here, we observed that the higher expression of miR-1307 in aggressive basal-like, HER2 and luminal B tumors (compare Fig. 1f) was globally associated with lower endothelial cell contents (Fig. 6a), reduced expression of angiogenesis-related genes (Fig. 6b) and a higher activity of hypoxia-associated gene signatures (Fig. 6c) when compared to the less aggressive luminal A subtype. Hence, endothelial cell content and angiogenesis were negatively correlated with both miR-1307-3p and more strongly with miR-1307-5p, whereas hypoxia was positively correlated with all 5′isomiRs of miR-1307 both across all tumors and within the PAM50 subtype luminal A (Fig. 6d). Together, these observations further support the relevance of miR-1307 in the regulation of tumor angiogenesis in breast cancer.

Since tumor angiogenesis and vasculature are potentially related to patient prognosis, we proceeded to investigate the prognostic value of the expression levels of both arms of miR-1307. For that purpose, we performed a Cox-PH analysis using PAM50 subtype and “primary diagnosis” (i.e., histological subtype) as strata and age at diagnosis as well as the expression of miR-1307-3p and miR-1307-5p as factors (Fig. 6e). We decided not to distinguish the expression of the two 5′isomiRs expressed from the 3p-arm due to their strong correlation, which might negatively affect the model if used as independent covariates. Here, we found a mild but highly significant association between the age at diagnosis and the overall survival probability of the patients. In addition, in line with the previously described oncogenic role of miR-1307-3p, we observed a significant association between high expression of miR-1307-3p and worse overall survival (OS, HR = 1.39, *p* = 0.03). In contrast, despite the substantial correlation of expression between the miRNAs expressed from the 3p- and the 5p-arm of pre-miR-1307 (see Fig. 1d), high expression of miR-1307-5p was associated with a better prognosis (HR = 0.83, *p* = 0.06). This is in line with the tumor-suppressive and anti-angiogenic roles of miR-1307-5p.

Next, we hypothesized that the observed differential arm usage might not be specific to pre-miR-1307 but may instead be regulated by a common mechanism for various pre-miRNAs. To this end, we investigated if any other pre-miRNAs with two expressed arms (*n* = 33) display similar patterns across all tumor samples regarding the relative expression (in percent) of their 5p arms as we had observed for miR-1307-5p across all tumors (Fig. 6f, Additional File 2: Table S2). Indeed, we observed significant *p* < 0.05) correlations with the arm usage of 30 other pre-miRNAs indicating a common regulatory mechanism. Notably, these correlations were partly negative, which suggests a complex, arm-independent regulation of arm usage in breast cancer.

In summary, our analyses of the TCGA BRCA cohort demonstrated that the increased expression of miR-1307-5p was associated with reduced tumor angiogenesis, presence of blood vessels as indicated by endothelial cell content, and tumor aggressiveness as indicated by our survival analyses. This function is contrasted by the previously described tumor-promoting functions of miR-1307-3p, emphasizing miRNA arm usage as a potential fine-tuning mechanism during cancer development and progression which might also be mediated by correlating alternative arm usage.

## Discussion

Angiogenesis as one of the hallmarks of cancer is a crucial prerequisite for tumor growth and metastasis [26]. However, the extensive inhibition of angiogenesis and tumor vasculature can paradoxically increase the tumor aggressiveness by promoting drug resistance and invasiveness through hypoxic signaling [27]. In this study, we demonstrated that miR-1307-5p, expressed in the breast cancer cells, acted as a potent suppressor of tumor angiogenesis, thereby reducing the formation of distant lung metastases in mice. In line with this, a high expression of miR-1307-5p was associated with an improved overall survival of breast cancer patients. Notably, both the oncogenic miR-1307-3p and the tumor-suppressive miR-1307-5p were co-upregulated in breast cancer tissue as compared to tumor-adjacent normal tissue.

The upregulation of miR-1307-5p in tumor tissue may initially appear counterintuitive, given its anti-angiogenic function, which could disadvantage the tumor growth. Hence, high expression of miR-1307 would be expected to undergo negative selection. However, transcriptional activation of pre-miR-1307 observed in aggressive breast cancer subtypes also results in increased levels of miR-1307-3p, which has been described to elicit oncogenic functions [22, 23]. Since the upregulation of both is most prominent in aggressive tumors, this may indicate that in these tumors, cells can cope better with impaired vasculature and the resulting hypoxic conditions. Alternatively, it might be associated with the tumor’s ability to bypass classical angiogenesis by the induction of vascular mimicry. Both scenarios have been previously described, specifically in basal-like breast cancer [28, 29]. Hence, in these tumors, the oncogenic effect of miR-1307-3p may outweigh the disadvantage mediated by miR-1307-5p. This hypothesis is in line with the data obtained from our mouse experiments, which indicate that the early induction of pre-miR-1307 was more efficient in blocking tumor growth compared to inducing expression after successful tumor formation. Along this line, the metastatic colonization was not impaired in our tail vein injection model. Breast tumors in patients are generally detected when they have already passed the time point of the angiogenic switch [30]. Consequently, the upregulation of miR-1307-5p in patient tumors may be an indication that the tumor-suppressive impact of the miRNA is less pronounced once tumors have reached a critical size and potentially alternative mechanisms to sustain their supplies.

From an evolutionary perspective, the concurrent upregulation of miR-1307-5p in highly proliferative tissue may represent a physiologically important negative feedback loop aimed at preventing uncontrolled proliferation by restricting angiogenesis. We and others have previously observed similarly paradoxical upregulation of tumor suppressive factors in aggressive tumors [18, 31], suggesting that this could be a relevant anti-cancer mechanism, which fails in case of successful tumor formation. Physiologically, such programs might be relevant during processes such as the menstrual cycle, wound healing, or breast tissue remodeling during pregnancy, when proliferation and angiogenesis are temporally required [32]. It can be speculated that this preventive mechanism has been overcome by aggressive tumors as they can cope better with impaired vasculature/hypoxia (e.g., basal breast cancer), potentially by vascular mimicry. Hence, these tumors would benefit from the oncogenic effects mediated by miR-1307-3p, such as enhanced proliferation and chemotherapy resistance [23]. At the same time, impaired angiogenesis and hypoxia might not counteract the progression of these advanced tumors anymore because they can grow aggressively also under hypoxic conditions [29].

Furthermore, the co-regulation of the arm usage patterns of pre-miR-1307 and other pre-miRNAs deserves further attention. Notably, we observed positive correlations between the relative abundance of miR-1307-5p and other miRNAs (e.g., miR-30e-5p, miR-127-5p, and miR-141-3p), which have been previously described to have tumor suppressive functions in breast cancer or other entities [33–37]. This potential co-regulation of various miRNAs further supports the hypothesis of an underlying physiologically important cellular program aimed at preventing tumorigenesis. Hence, further studies addressing the causative mechanisms are needed to pave the way for novel therapeutic strategies, that could potentially counteract tumorigenesis by simultaneously shifting the balance towards a set of miRNAs with complementary tumor-suppressive functions.

## Conclusions

Taken together, our findings establish miR-1307-5p as a novel regulator of angiogenesis in breast cancer. At the same time, our results underscore the importance of future research focusing on the regulation of miRNA arm selection in cancer. Uncovering the unknown underlying mechanisms may provide a foundation for innovative therapeutic strategies aimed at counteracting tumor progression by coordinated suppression of multiple oncogenic traits through a shift in the arm-usage pattern towards the tumor-suppressive miRNA species.

## Materials and methods

Detailed descriptions of the experimental procedures can be found in Additional File 3 [18, 38–61].

### Processing of data from TCGA-BRCA cohort

TCGA-BRCA mRNA and miRNA isoform quantification data aligned to the GRCH38 build (hg38) were downloaded from the GDC harmonized database using the Bioconductor R package TCGA biolinks (version 2.12.6). Meta-data and information on potential confounders were gathered from different sources as described previously [18]. Hypoxia scores were downloaded from cBioportal in June 2021 [62] (Additional File 2: Table S3).

Batch-corrected miRNA isoform (= isomiR) quantification data were obtained from GEO (Gene expression omnibus, GSE164767 [63]) and further processed as detailed in Additional File 3. Briefly, isomiR expression data were collapsed to obtain 5′isomiR (Additional File 2: Table S4) and miRNA arm level expression (Additional File 2: Table S5), mRNA expression data was cleaned and tpm (transcripts per million) expression values were calculated (Additional File 2: Table S6), and patient samples were filtered as detailed in Additional File 3.

Uncollapsed expression values, i.e., expression values of all possible isomiRs derived from hsa-miR-1307 were used to generate the overview heatmap (Additional File 1: Fig. S1). Here, the log10(x + 1) mean expression (tpm values) of each isomiR was plotted based on the respective start and end positions.

For the combined analyses of mRNA and miRNA data, only samples for which isomiR and mRNA data were available were used, resulting in 1085 samples (523 LumA, 187 LumB, 161 Basal, 78 Her2, 34 Normal-like, 102 Normal). Normal-like tumors were excluded from the analyses due to the low sample size. Confounder data can be found in Additional File 2: Table S7.

To estimate the association between isomiR levels and angiogenesis-related, the Angiogenesis geneset was downloaded from the Hallmarks collection of the MSigDB database (version: v2022.1.Hs updated August 2022, downloaded October 5th, 2022) [38]. Genes for which no expression (i.e., 0 tpm) was measured in any patient were excluded. Gene expression was z-scaled for each patient over all genes and the median z-score over all genes per patient is referred to as the “activity score” (Additional File 2: Table S8).

### Cell culture: origin of cell lines, media used, subcultivation protocols

Adherent cells were cultivated at 37 °C and 5% CO_2_ in a humidified incubator until 80% of confluency. HUVEC cells were cultured in Endopan3 media with supplements (Pan-Biotech) and 2% BSA. MDA-MB231 wild type (wt) and stable cells (origin: ATCC HTB-26) were cultured in RPMI-1640 (Gibco, #21,875,034) supplemented with 10% FBS (Gibco) and 1% P/S. BT549 wt and stable cells (origin: ATCC HTB-122) were cultured in RPMI-1640 supplemented with 10% FBS, 1% P/S, and 0.1% insulin (Sigma-Aldrich).

### Generation and validation of stable pre-miR-1307 overexpression cell lines

Cell lines with stable doxycycline-inducible overexpression of pre-miR-1307 or one of two different *C. elegans* control pre-miRNAs were generated by retroviral transduction as previously described [18, 39, 40] (see Additional File 3).

For small RNA sequencing, cells were seeded in triplicates into 6-well-plates and transgene expression was induced for 48 h with doxycycline. RNA was extracted using the Qiagen miRNAeasy kit (Cat. No.: 217084) according to the manufacturer’s protocol. Samples were prepared for small RNA sequencing using the NEBNext system following the manufacturer’s instructions. Sequencing was performed on a NovaSeq6000 device running a S1 cell for 50 bp paired-end sequencing. Further details are described in Additional File 3. Reads per million (rpms) were calculated and isomiRs were filtered for a summarized expression > 15 rpm over all samples in order to exclude artifacts. Read counts were then summed up to the miRNA level as described above for the TCGA data and log2 transformed. Differential expression analysis was performed on the raw read counts using DESeq2 (version 1.38.0) and employing the apeglm function (version 1.20.0) [64]. isomiRs with a median of less than 5 read counts across all samples were excluded from the differential expression analysis. Sequencing results are available on GEO (GSE227354) [65], whereas the R code for differential expression analysis and calculation of rpm values is available in our GitHub repository (https://github.com/Klammeraffe7/pre-miR-1307) [66].

### Cell proliferation assay

Cell proliferation assays were conducted as detailed in Additional File 3. The median value of six technical replicates was used for each biological replicate. Statistical analysis was performed using a two-tailed paired *t*-test at the final time point (6 days after transfection).

### Xenograft experiments

Experimental details are provided in Additional File 3; all experiments were performed using stably transduced MDA-MB-231 cells.

Cells were injected in 30 μL PBS:Matrigel under isoflurane anesthesia into the 3rd mammary gland fat pad of NSG mice (*n* = 5–6/group) kept at a 12-h light–dark cycle with unrestricted Kliba 3307 diet and water. Doxycycline was given in drinking water starting 7 days post-injection. Twice a week, the tumor size was measured by a caliper in two dimensions until the tumor reached 1 cm in one diameter or until an alternative predefined humane endpoint was reached. Lungs and primary tumors were collected for further analyses as detailed below. The animal experiment was licensed under G288/14 by the local regulatory authorities (regional council, Karlsruhe, Germany).

To analyze the impact of pre-miRNAs on tumor formation, cells were orthotopically injected into the mammary fat pat of NOG mice. One week after injection, when tumors were still not palpable, doxycycline was given in drinking water. Sixty-nine days after injection, the mice were sacrificed and tumor weights were measured.

For the analysis of the cells’ potential for metastatic colonization, cells were incubated with doxycycline for 72 h to induce transgene expression and injected into the tail vein of female NOG mice (*n* = 6/group). 48 h after inoculation, doxycycline was given in drinking water, and mice were sacrificed 3 weeks later. Lungs were excised, photographed, and quantified metastatic nodules based on the GFP signal. The data are presented as averages ± SEM. The latter animal experiments and methods were approved by the Weizmann Institutional Animal Care and Use Committee.

### Quantification of xenograft tumor vascularization by CD31 staining (IF)

Acetone was used to fix 5 μm thick cryo-preserved lung sections for 10 min, then sections were rehydrated with PBS. 1% ELISA BSA was applied for 1 h to reduce unspecific background staining. Then, tissues were subjected to rat anti-mouse CD31 antibody (BD Pharmingin, #553,370, 1:50 diluted in blocking solution). Donkey anti-rat IgG Cy3 (Jackson, Dianova, 1:200 diluted in blocking solution) was used as a secondary antibody. The preparations were counterstained with Hoechst (4 µg/ml) and mounted with a DAKO mounting medium.

### Quantification of micrometastases in the lungs from orthotopic xenograft experiments

Eight random 10-μm sections from each paraffin-embedded lung were collected and pooled for the analysis of micrometastasis by the Alu PCR method previously described in Funakoshi et al. (2017) [47]. Reagents and assay details are summarized in Additional File 2: Table S9 and the experimental details are described in Additional File 3. Data acquisition and raw data analysis were performed using QuantStudio PCR Systems (Applied Biosystems). For analysis, the median of the technical triplicates per sample was used. With the help of a calibration curve (Additional File 1: Fig. S4), the human DNA amount within the total DNA isolated from mice lung were determined for the identification of micro metastasis in percent.

For GFP-IHC-based detection of micrometastases, lung sections were taken on PLL slides and stained with an anti-GFP antibody as detailed in Additional File 3. Successful induction of GFP expression was demonstrated by IHC staining of the primary tumors (Additional File 1: Fig. S2). The preparations were counterstained with 0.5 mg/mL Harris hematoxylin, mounted with mounting media EUKITT®.

### IHC and IF tissue section scanning and analysis

Miscellaneous slide scanner Zeiss Axio Scan 7 (Zeiss Microscopy) was used to scan the stained tissue sections. Images were acquired by the accompanying software ZEN (blue edition) and scanned images were converted into Tiff (for IHC image analysis) or OME.tiff (for IF image analysis) format. The imaging software FIJI [67] was used for quantitative analyses as detailed in Additional File 3.

### Computational estimation of endothelial cell content in TCGA-BRCA samples

Endothelial cell content in TCGA-BRCA samples was inferred based on DNA methylation using the Houseman algorithm [54] implemented in the RnBeads R package and RNA expression data using MCPcounter [56]. Methodological details are described in Additional File 3. The results of the estimation of endothelial cell contents per sample are summarized in Additional File 2: Table S10 and S11.

### Sprouting assay

The sprouting protocol was adapted from Tetzlaff and colleagues [57, 58]. Experimental details are described in Additional File 3. Conditioned media was collected from BT-549 or MDA MB-231 cells that were transfected with isomiR mimics or stably expressing pre-miR-1307. Cells were incubated for 2 (transfected cells) or 3 days (stable cells) at 37 °C and 5% CO_2_. Conditioned media were collected in 2% FBS for 24 h.

For the sprouting assay, human umbilical vein endothelial cells (HUVECs) were cultured in Endopan 3 media containing 3% FBS and supplements at 37 °C and 5% CO_2_ until they reached full confluency. Images of the spheroids were acquired with the Axiovert25 microscope with 5 × magnification. Image analysis was performed using ImageJ to determine the number and the average length of sprouts. Ten spheroids per condition were averaged for the analysis.

### Secretome analysis by mass spectrometry

For MS-based analysis of secreted proteins, MDA-MB-231 cells were transfected with miRNA mimics for 48 h. The cells were washed twice with DPBS to remove residual FBS and secretomes were collected for 16 h in a serum-free medium. Protein digestion and clean-up was performed as detailed in Additional File 3 along with further experimental details about the LC–MS/MS analysis. Peptide and protein identification and quantification from DIA raw data was performed with the Biognosys software Spectronaut (version 15.5) in directDIA mode.

The mass spectrometry proteomics data were deposited to the ProteomeXchange Consortium via the PRIDE partner repository [60] with the dataset identifier PXD041087 [68]. Protein quantitation data were log2 transformed (Additional File 2: Table S1) and further processed as described in Additional File 3. Filtered data were used to generate a heatmap and a Venn diagram which shows the number of significantly changed proteins for each miRNA (*p* value < 0.05). R, pheatmap (https://CRAN.R-project.org/package=pheatmap) and the eulerr package (https://CRAN.R-project.org/package=eulerr, version 7.0.0) were used for this purpose.

### Cox-PH regression analysis

To evaluate the prognostic value of miR-1307-3p and miR-1307-5p expressions in breast cancer, a multivariate Cox Proportional Hazards (Cox-PH) regression model was used. The model was generated based on the overall survival (OS) and end-point information of 933 breast cancer patients filtered as detailed above from the TCGA BRCA dataset [61]. Further details are described in Additional File 3.

The log2-transformed rpm of the 3p- and the 5p-arm of pre-miR-1307 as well as the age of diagnosis for each patient were assigned as covariates while primary diagnosis and molecular subtype information of patients were used for stratification in the model. 95% confidence intervals (CI) for the hazard ratios (HR) were used and CI values, HRs, and *p*-values for each estimate were displayed in a forest plot. All analyses were performed using the survival (v.3.4.0) library and the forest plot was generated using the forestmodel (v.0.6.2) library in R (v.4.1.3).

### Arm usage heatmap

The heatmap visualizing miRNA arm usage was generated based on the TCGA expression data of miRNAs in R. Expression of the arms from the isomiRs were summed per miRNA and patient. miRNAs that did not express both arms in all patients were filtered out. The percentage of the 5p arm was calculated by dividing its expression by the total expression from both arms (Additional File 2: Table S2). The Spearman correlation was calculated and *p*-values were adjusted with Benjamini Hochberg correction where comparisons with an adjusted *p*-value ≤ 0.01 were considered as significantly correlated. The heatmap was created using the ComplexHeatmap package and annotated using the TCGA metadata described above.

### Data analysis and visualization

Analyses in R were performed using version 4.2.1 if not indicated differently. R packages used for analysis and visualization are summarized in Additional File 3.

## Supplementary Information

Additional file 1: Figures S1-S4. Fig. S1 – Heatmap of isomiR expression in TCGA BRCA. Fig. S2 – GFP IHC images of mouse tumors. Fig. S3 – Ki67 staining of mouse tumors. Fig. S4 – Calibration curve for Alu-PCR

Additional file 2: Tables S1-S11. Tab. S1 – Protein abundance values, log2 fold changes and p-values obtained from the secretome analysis for significantly regulated proteins. Tab. S2 – Percentages of 5p expression of miRNAs with two expressed arms. Tab S3 – TCGA hypoxia scores. Tab S4 –5'isomiR collapsed isomiR expression in TCGA BRCA samples. Tab. S5 – Arm-collapsed isomiR expression in TCGA BRCA samples. Tab. S6 – mRNA expression values of TCGA BRCA samples. Tab. S7 – Confounder information on TCGA BRCA samples. Tab. S8 – Activation scores for TCGA BRCA genes. Tab. S9 – Primers, probe and experimental details for TaqMan based Alu-qPCR. Tab. S10 – Estimated endothelial cell content of TCGA BRCA samples based on MCPcounter. Tab. S11 – Estimated endothelial cell content of TCGA BRCA samples based on RnBeads

Additional file 3: Supplementary Methods. Processing of data from TCGA-BRCA cohort - Generation and validation of stable pre-miR-1307 overexpression cell lines - Xenograft experiments - Quantification of micrometastases in the lungs from orthotopic xenograft experiments - Computational estimation of endothelial cell content in TCGA-BRCA samples - Sprouting assay - Secretome analysis by Mass Spectrometry - COX-PH regression analysis - Data analysis and visualization

## Data Availability

Small RNA sequencing data of MDA-MB-231 cells overexpressing pre-miR-1307 or control pre-miRNAs are available at GEO (GSE227354) [65]. Raw data of the Mass-Spec-based secretome analysis are available at the PRIDE partner repository [60] with the dataset identifier PXD041087 [68]. Analyzed data are provided in Additional File 2: Table S1 and S2 and the underlying code is published on GitHub (https://github.com/Klammeraffe7/pre-miR-1307) [66]. Cell lines will be made available upon request.

## Abbreviations

CI: Confidence interval
Cox-PH: Cox proportional hazards
DIA: Data-independent acquisition
DOX: Doxycycline
FBS: Fetal bovine serum
GFP: Green fluorescent protein
HER2: Human epidermal growth factor receptor 2
HR: Hazard ratio
HUVEC: Human umbilical vein endothelial cells
IF: Immunofluorescence
IHC: Immunohistochemistry
isomiR: Isoform of a microRNA
LC–MS/MS: Liquid chromatography–tandem mass spectrometry
LumA: Luminal A
LumB: Luminal B
miR: MicroRNA
NOG: NOD/Shi-scid/IL-2Rγnull
NSG: NOD scid gamma
OS: Overall survival
PAM50: Prediction Analysis Of Microarray 50
pre-miR: Precursor-microRNA
TCGA-BRCA: The Cancer Genome Atlas-Breast Invasive Carcinoma
