# Supplementary material for: Selective arm-usage of pre-miR-1307 dysregulates angiogenesis and affects breast cancer aggressiveness
Source: BMC Biol. 2025 Jan 23;23:25. doi: 10.1186/s12915-025-02133-x (PMC11756181; doi:10.1186/s12915-025-02133-x)
Supplement: Supplementary file 1 — Additional file 1: Figures S1-S4. Fig. S1 – Heatmap of isomiR expression in TCGA BRCA. Fig. S2 – GFP IHC images of mouse tumors. Fig. S3 – Ki67 staining of mouse tumors. Fig. S4 – Calibration curve for Alu-PCR [file 12915_2025_2133_MOESM1_ESM.docx]

**Additional File 1: Supplementary Figures**


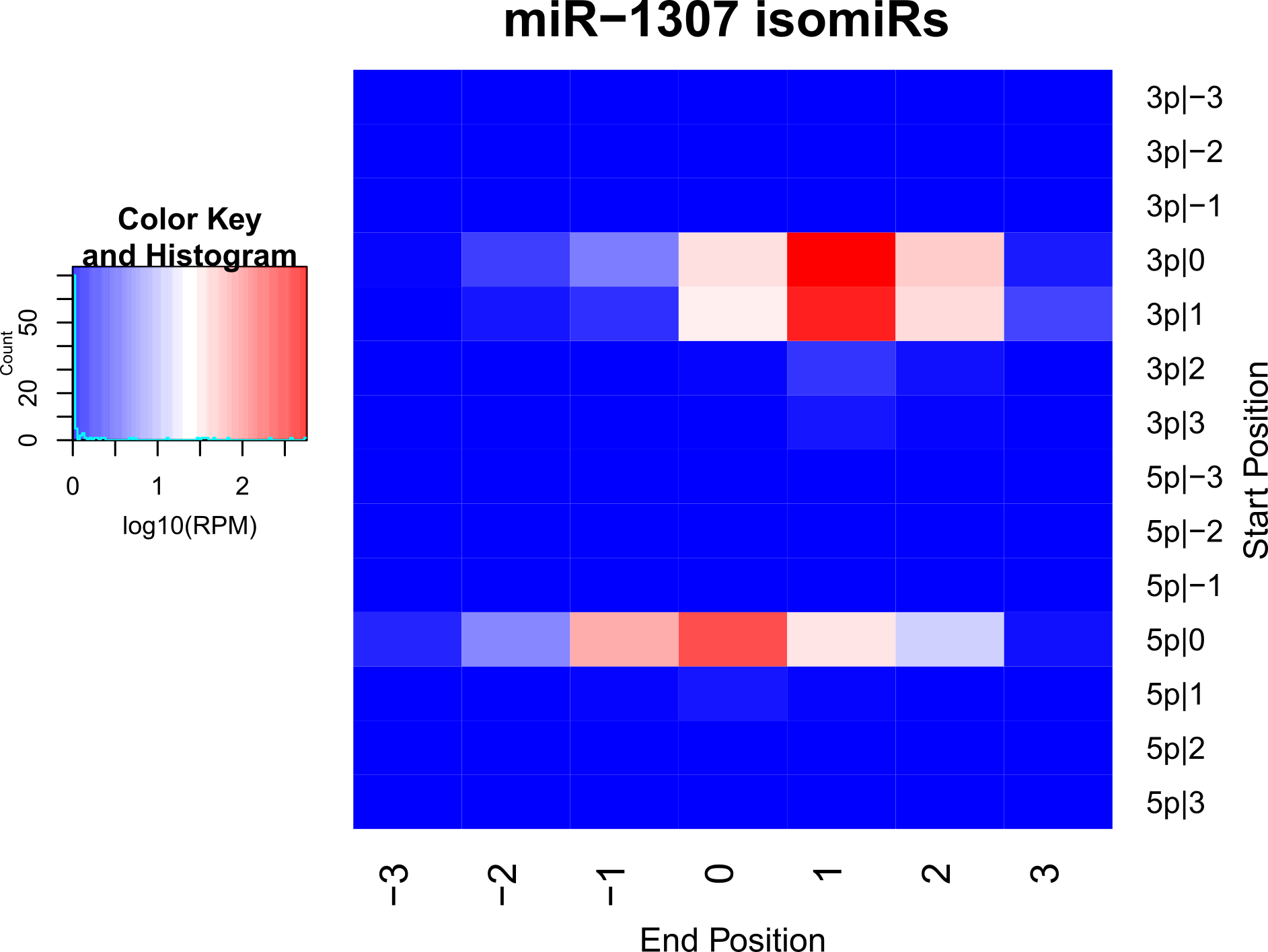


**Figure S1.** Mean expression of isomiRs derived from pre-miR-1307 across all TCGA samples based on their start and end positions. Log10(x+1) expression in rpm is depicted. miR-1307-5p|0, -3p|0 and -3p|+1 are the predominant 5’ variants with varying 3’ end variants. The most abundant isomiRs are hsa-miR-1307-3p|0|1|, hsa-miR-1307|1|1| and hsa-miR-1307-5p|0|0|. The majority of possible isomiRs is not or only very lowly expressed.
The synthetic miRNA mimics used for *in vitro* experiments in this study represent the |0 variant at the 3’ end.

**Figure 3.** Ki67 staining of pre-miR-1307 overexpressing mouse tumors. MDA-MB-231 cells overexpressing the indicated pre-miRNA were injected orthotopically into the mammary fat pad of NSG mice. The mice were sacrificed when the humane endpoint based on tumor size was reached and tumor slices were stained for Ki67. The number of Ki67 positive cells per mm² tumor tissue was determined using an in-house ImageJ macro.


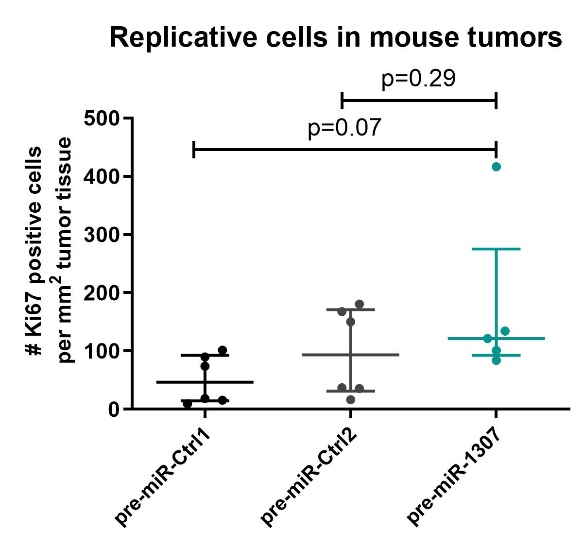


**Figure S2.** GFP IHC images. Stable MDA-MB-231 cells were induced with doxycycline 2 weeks after injection to mice. Each stable cell line inducible miRNA construct has GFP expression site which is inducible with doxycycline. a) Images shows GFP antibody treated (right) and untreated tumor tissues. Tissues were also counterstained with nuclear hematoxylin staining. Brown areas represent GFP positive, and blue areas represent hematoxylin positive staining. **b)** Representative IHC staining images for GFP expression in tumor from each group. For imaging Zeiss Axio Scan 7 from Zeiss Microscopy was used with the software Zeiss (blue edition). Scale bar represents 200 μm. Original images were captured at 10X magnification.


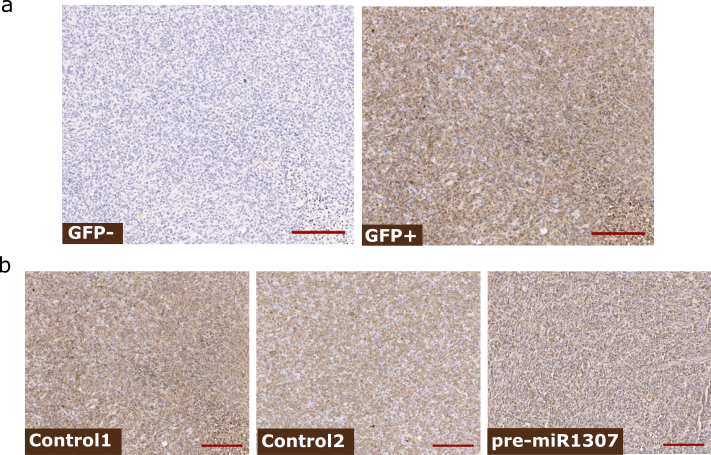

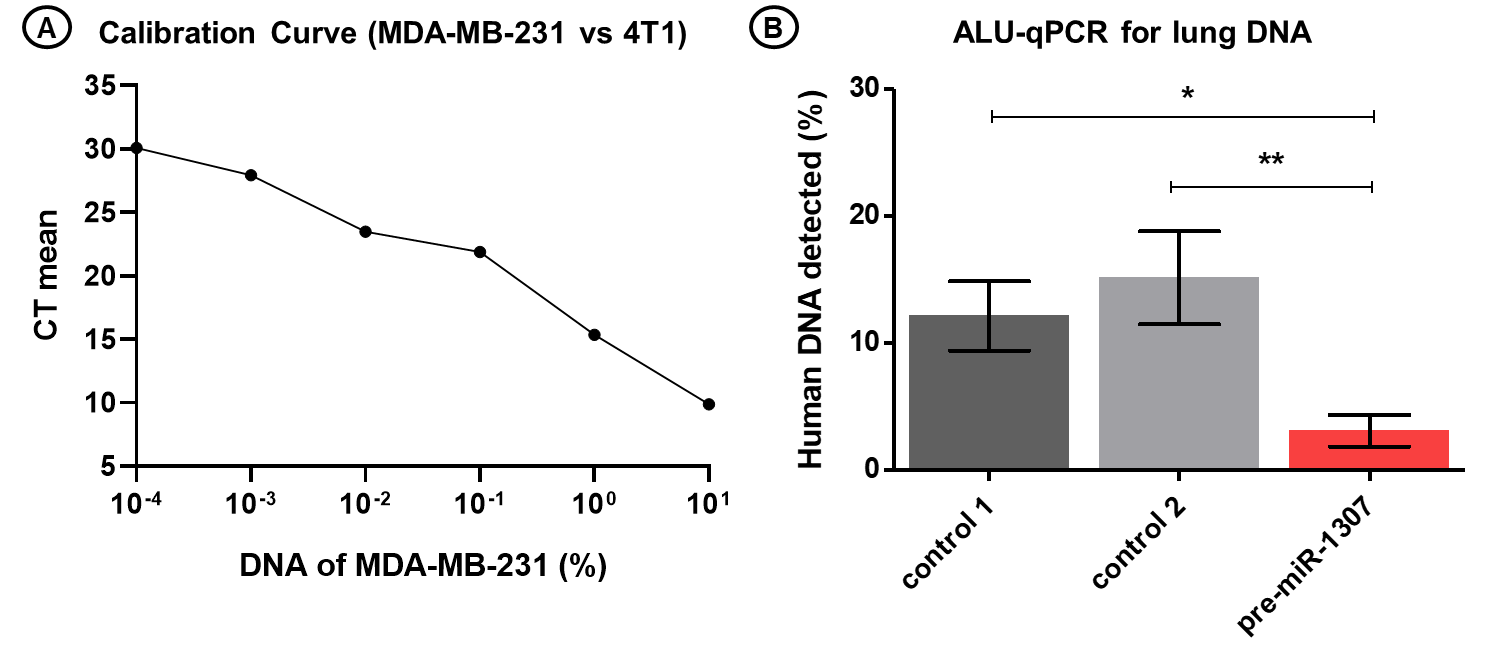


**Figure S4.** Calibration curve showing how CT value changed with the respective amount of human DNA mixed with rodent DNA. DNAs of human MDA-MB-231 cells and mouse 4T1 cells were mixed. The amount of MDA-MB-231 cell’s DNA in the mixture were represented in the x axis in log scale. Corresponding CTmean values for each technical replicate (n=3) were demonstrated in the y axis. Regression line (blue) were drawn and the equation was used to estimate Human DNA amount in the lungs.
