# Supplementary material for: Selective arm-usage of pre-miR-1307 dysregulates angiogenesis and affects breast cancer aggressiveness
Source: BMC Biol. 2025 Jan 23;23:25. doi: 10.1186/s12915-025-02133-x (PMC11756181; doi:10.1186/s12915-025-02133-x)
Supplement: Supplementary file 3 — Additional file 3: Supplementary Methods. Processing of data from TCGA-BRCA cohort - Generation and validation of stable pre-miR-1307 overexpression cell lines - Xenograft experiments - Quantification of micrometastases in the lungs from orthotopic xenograft experiments - Computational estimation of endothelial cell content in TCGA-BRCA samples - Sprouting assay - Secretome analysis by Mass Spectrometry - COX-PH regression analysis - Data analysis and visualization [file 12915_2025_2133_MOESM3_ESM.docx]

**Supplementary Methods**

*Processing of data from TCGA-BRCA cohort*

Batch corrected miRNA isoform (= isomiR) quantification data were obtained from GEO (Gene Expression Omnibus, GSE 164767) using isomiRs with a median expression above 15 rpm amongst all samples (GSE164767_TCGA-BRCA_corrected_all_median15.txt.gz) and collapsed to 5’isomiR expression values. To this end, we summed up the read counts of all 3’ isomiRs for a given 5’isomiRs per sample. Expression of each 5’isomiR is hence defined as the sum of all respective 3’isomiRs. 246 different 5’isomiRs (including the canonical version) were obtained after this collapsing step (Supplementary Table 2). To analyze miRNA arm usage, we further summed up all isomiR reads per arm to consider only the respective 5p or 3p arm of a miRNA and refer to this version as “arm-collapsed” (Supplementary Table 3). The resulting dataset contained data for 179 arm-collapsed isomiRs.

Patient samples were further filtered to reduce the influence of technical variations: seven metastatic samples, 20 samples with a tumor purity of less than 40% and 14 samples derived from male patients were excluded from the analysis. 19 of the 1162 samples, which were contained in the dataset, had been sequenced two or more times. Here, we kept the sample with the highest sequencing depth resulting in 1102 patient samples for which isomiR data were available. For the arm-collapsed isomiRs, only those patients were further considered, for which also mRNA data were available, resulting in 1085 patients. The above mentioned steps were performed in R, version 4.2.2.. The code to reproduce these steps can be found in the git repository in the script “TCGA_BRCA_isomiR_collapse.Rmd”.

For the mRNA data, substantial efforts were made to reduce the effect of technical influences during the sequencing process on the analysis. The following steps were performed in R. Read counts from the htseq-count analysis were provided for 1222 BRCA patients and 60483 genes by the GDC. Individual files for each sample were compiled to a table containing reads for all genes and patients and annotated with hgnc symbols using R (version 4.2.0) and biomaRt (version 2.52.0). Code is available in the git repository as “TCGA_mRNA_htseqcount_data.R”. When using the TCGA-mRNA data for a variety of analyses, we realized that for certain patients up to ca. 65% of reads were assigned to a group of 16 mitochondria-associated genes (MT-CO3, MT-CO2, MT-CO1, MT-ND4, MT-RNR2, MT-ATP6, MT-CYB, MT-ND1, MT-ND3, MT-ND2, MT-ATP8, MT-ND4L, MT-ND6, MT-ND5, MT-RNR1 and MT-TP). To our knowledge, this is unlikely to reflect the biology of tumor development. We therefore decided to exclude these MT-genes completely from the analysis. As described for the miRNA sequencing, we again excluded seven metastatic samples and focused on samples from female patients by excluding 14 male samples as well as 23 samples with a tumor purity lower than 40%. The organizational setup of the TCGA consortium included sequencing of samples of different entities on the same plate. For some samples, this implied that only very few samples of one entity were sequenced on one plate. To minimize the high variation that came with sequencing on different plates to a reasonable level, we excluded 25 samples that came from sequencing plates containing less than eight BRCA samples. Lowly expressed genes were filtered out using the edgeR (version 3.36.0) cpm functionality and removing those genes, for which the sum of reads over all patients was lower than half the number of patients analysed. Read counts for the remaining 1153 samples were converted to transcripts per million (tpm) using the GenomicFeatures (version 1.46.1) package and downloading the gencode.v22 annotation from https://www.gencodegenes.org/. Finally, only those genes were kept for further analyses for which HGNC symbols were provided leaving 14650 genes for further analysis. For one sample (TCGA-A7-A0DC-01A-11R-A00Z-07), removing genes without HGNC symbol resulted in a loss of almost half of all reads and thus this sample was removed from further analyses (Supplementary Table 4). Corresponding code can be found in the “TCGA_BRCA_mRNA_removeMTgenes_TPMcalculations.Rmd” script in the git repository.

For further analyses, only samples for which isomiR and mRNA data were available were used, resulting in 1085 samples (523 LumA, 187 LumB, 161 Basal, 78 Her2, 34 Normal-like, 102 Normal). Normal-like tumors were excluded from the analyses due to low sample size. Confounding variables for the samples were derived as described previously (Ibing et al., 2021) and using the TCGAbiolinks R package (version 2.24.3) to obtain BRCA subtype information. A summary for the 1085 final samples can be found in Supplementary Table 5.

To estimate the association between isomiR levels and angiogenesis-related or respectively hypoxia-associated processes, the Angiogenesis and Hypoxia gene sets were downloaded from the Hallmarks collection of the MSigDB database (version: v2022.1.Hs updated August 2022, Downloaded October, 5th 2022) [38]. Gene expression in tpm for each gene in the gene set was obtained from the TCGA-BRCA mRNA data for all tumor samples or the respective subtype. Genes for which no expression was measured in any patient were excluded. Gene expression was z-scored for each patient over all genes and the median z-score over all genes per patient is referred to as activity score. Corresponding code is deposited in the git repository as “TCGA_activity_scores.Rmd” script. Results can be found in Supplementary table 1.

*Generation and validation of stable pre-miR-1307 overexpression cell lines*

Stable cell lines overexpressing the pre-miR-1307 or one of two different *C.elegans* control pre-miRNAs were generated by retroviral transduction as previously described [18]. Briefly, the pre-miRNA was cloned into the retroviral RT3GEPIR vector [39] and co-transfected with the pMD2.G plasmid (VSV-G envelope, Addgene # 12259) and the pHIT60 plasmid (gag/pol packaging; [40]) into HEK293FT cells. After 24 h, the supernatant was collected, centrifuged and filtered and added to target cells together with Polybrene (10 µg/mL, Merck, Germany) for 24 hours. Medium was then exchanged to fresh full growth medium supplemented with 2 µg/mL puromycin (Sigma) to select for successfully transduced cells.

For small RNA sequencing, MDA-MB-231 cells containing either the pre-miR-1307 or one of two different *C.elegans* control pre-miRNAs were seeded in triplicates into 6-well-plates and transgene expression was induced for 48h with doxycyclin. RNA was extracted from samples using the Qiagen miRNAeasy kit (Cat. No. 217084) according to the manufacturer’s protocol. Samples were prepared for small RNA sequencing using the NEBNext Small RNA Library Prep Set for Illumina (Cat. No. E7330S) and the NEBNext Multiplex Oligos for Illumina (Index Primers Set 2, Cat. No. E7500S) following the manufacturer’s instructions. Samples were cleaned after PCR amplification with the Monarch PCR & DNA Cleanup Kit (NEB, Cat. No.: #T1030) and Agencourt AMPure XP Beads (Beckman Coulter, Inc. #A63881) were used two times for size selection as detailed in the library preparation kit and the resulting size distribution was analyzed using Tapestation. Sequencing was performed on a NOVASEQ6000 device running a S1 cell for 50 bp paired end sequencing. Forward reads were used for analysis. Read counts were mapped to the genome using the sRNA mapper, an in-house pipeline which runs on the DKFZ HUSAR platform and maps the reads using bowtie (version 0.12.9, [41]). Samtools (version 1.14) [42, 43] was used to created index files and the bedtools multicov functionality (version 2.27.1) [44, 45] was used for intersection of mapped reads with an in-house build isomir gff file [46]. Reads per million (rpms) were calculated from the raw readcounts. isomiRs were filtered for a summarized expression > 15 rpm over all samples to exclude artifacts. Read counts were then summed up to the miRNA level as described above for the TCGA data and log2 transformed. Differential expression analysis was performed on the raw read counts using DESeq2 (version 1.38.0) and employing the apeglm function (version 1.20.0). isomiRs with a median of less than 5 read counts over all samples were excluded from the differential expression analysis. Sequencing results are available on GEO (GSE227354), R code for differential expression analysis and calculation of rpms is available in the git repository as “MDA_MB_231_Formating_miRNA_seq_counts_rpms.Rmd” and “MDA_MB_231_DESeq2.Rmd”, respectively.

Xenograft experiments

To analyze tumor growth, 3x10^6^ MDA-MB-231 cells stably overexpressing pre-miRNA (pre-miR-1307 or two different pre-miRNA negative controls) were injected in 30 μL PBS:Matrigel (Corning, Bedford, USA, growth factor reduced, 1:1, v/v) under isoflurane anesthesia into the 3rd mammary gland fat pad of 6-7 week-old female NSG mice (n=5-6/group) kept at a 12 h light–dark cycle with unrestricted Kliba 3307 diet and water. Seven days post injection, when tumors were already palpable, doxycycline (1 mg/mL in drinking water supplemented with 5% saccharose) was given. The Kliba 3307 and the drinking water including ingredients were replaced once every week throughout the study. Twice a week, the tumor size was measured by caliper in two dimensions. The weight of mice was recorded once weekly. Mice were followed up for 12 weeks and sacrificed once the tumor reached 1 cm in one diameter or if an alternative predefined humane endpoint was reached. Lungs and primary tumors were collected for further analyses detailed below. The animal experiment was licensed under G288/14 by the local regulatory authorities (regional council, Karlsruhe, Germany).

To analyze the impact of pre-miRNAs on tumor formation, 3x10^6^ stably transduced MDA-MB-231 cells were orthotopically injected into the mammary fat pat of NOG mice. One week after injection, when tumors were still not palpable, doxycycline (1 mg/mL in drinking water supplemented with 5% saccharose) was given. Throughout the study, the water was replaced with fresh DOX every 48h. 69 days after injection, the mice were sacrifized and tumor weights were measured.

For the analysis of the cells’ potential for metastatic colonization, stably transduced MDA-MB-231 cells were incubated with doxycycline for 72h to induce transgene expression. 2 × 10^4^ cells with inducible overexpression of GFP along with pre-miR-Ctrl and pre-miR-1307 were injected into the tail vein of female NOG mice (n = 6/group). 48h after inoculation, doxycycline (1 mg/ml in 5% saccharose) was given in drinking water. Throughout the study, the water was replaced with fresh DOX every 48h and mice were sacrificed 3 weeks later. Lungs were excised, photographed and metastatic nodules quantified based on the GFP signal. Shown are representative fluorescence images of lungs of each group. The data are presented as averages ± SEM. The latter animal experiments and methods were approved by the Weizmann Institutional Animal Care and Use Committee.

*Quantification of micrometastases in the lungs from orthotopic xenograft experiments*

By Alu-PCR

Lungs embedded in paraffin were checked for micrometastasis by Alu PCR method previously described in Funakoshi et al., 2017 [47]. Eight random 10 μm sections from tumor tissues embedded in paraffin were collected and pooled per specimen/biological replicates. Then, genomic DNAs from formalin-fixed paraffin-embedded tissues were purified using QIAamp DNA FFPE Tissue Kit (Qiagen, Hilden, Germany). DNA concentrations were diluted to 15 ng/μL for further steps. Reagents and assay details are summarized in Supplementary Table 7. Data acquisition and raw data analysis were performed using QuantStudio PCR Systems (Applied Biosystems). For analysis, the median of the technical triplicates per sample was used. For generating a CT vs. known DNA calibration curve, isolated DNAs of human MDA-MB-231 and mouse 4T1 cell lines were mixed in certain amounts and diluted serially (10^1^-10^-4^ % MDA-MB-231 DNA within 4T1 DNA). With the help of this calibration curve (Supplementary Figure 1), the relationship between the Ct value and the human-mouse DNA ratio was identified. Based on this, human DNA amount within the total DNA isolated from mice lung were determined for the identification of micro metastasis % based on the Ct values.

By GFP-IHC

Tumor and lung sections were taken on PLL slides. Slides were rehydrated by water and PBS. Subsequently, antigen retrieval was done by applying 10 μM sodium nitrate pH 6.0 with 0.05% Tween20 for 20 minutes to unmask necessary epitopes on the tissue. Then, 3% H2O2 in PBS was introduced for 10 minutes to block endogenous peroxidase activity. After 10% goat serum was applied for 30 minutes to reduce unspecific background staining, tissues were subjected to rabbit anti GFP (Abcam, ab290, 1:500 diluted in PBS). Then, goat anti-rabbit IgG HRP (Jackson, 1:400 diluted in PBS) was used as a secondary antibody. Diaminobenzidine (DAB, Sigma Aldrich, D3939-1 set) was then used for 1 minute as the substrate for localizing the antibody binding. The preparations were counterstained with 0.5 mg/mL Harris hematoxylin, mounted with mounting media EUKITT®.

*Computational estimation of endothelial cell content in TCGA-BRCA samples*

To infer endothelial cell content in TCGA-BRCA samples based on methylation data, Illumina HumanMethylation450 data (idat files) were downloaded from GDC (Genomic Data Commons Data Portal, https://portal.gdc.cancer.gov). Reference data for six immune cell types (CD4+ T-cells, CD8+ T-cells, CD14+ monocytes, CD19+ B-cells, CD56+ NK-cells) were available from (Andrew E Jaffe (2021). FlowSorted.Blood.450k: Illumina HumanMethylation data on sorted blood cell populations. R package version 1.32.0). Additional datasets used as reference datasets were downloaded from GEO: human mammary fibroblast, human mammary epithelial cells, human mammary endothelial cells (n=2 each, GSE74877, [48]), human breast cancer cell lines (MCF7, AU565, MDA-MB-231; GSE68379, [49]). All raw data were preprocessed using the SeSAMe package [50] using linear dye-bias correction (dyeBiasCorr) and background subtraction using noobsb. Further pre-processing was done using the RnBeads R package [51, 52]. We filtered out probes having a SNP overlapping with the C nucleotide of the CG site and a MAF > 0.01 (dbSNP 150, n=32,092), as well as probes when the last 3 bases in their target sequence overlap with a SNP (MAF >0.05, as defined in RnBeads, n = 7,770). Additionally, we removed cross-hybridizing probes (n = 27,592), according to Chen et al. [53] and 1326 non-CpG probes (as defined in RnBeads). The final dataset consisted of 927 samples and 416797 probes. Cell type composition was estimated using the Houseman algorithm [54] implemented in the RnBeads R package, using the settings inference.max.cell.type.markers = 100000, inference.top.cell.type.markers = 500. Cell composition results were proportionally adjusted to a sum of 100%.

As an orthogonal approach, we implemented a deconvolution approach based on RNA expression. To this end, complete analysis was performed on R/Bioconductor software (R 4.1.0) by applying the unified workflow from the immunedeconv R package (v 2.1.0) [55], using marker gene- based (MCPcounter) [56], to infer the relative cellular composition for each patient based on the non-log transformed and TPM-normalized gene expression data (Supplementary Table 4). The results of the estimation of endothelial cell contents per sample are summarized in Supplementary Table 8 and 9.

*Sprouting assay*

The sprouting protocol was adapted from Tetzlaff and colleagues [57, 58]. Methocoel solution was prepared by first autoclaving 6g methyl cellulose and adding 250mL pre-warmed (60°C) Endopan 3 basal media. After 20min of stirring, another 250mL of Endopan 3 basal media was added and the solution was stirred overnight at 4°C. Finally the solution was aliquoted and then centrifuged at 5000 x g at RT for 2h.

The collagen media was prepared using 4mL of the collagen stock solution mixed with 0.5 mL 10x media 199 on ice. The pH was set by dropping cold 0,2M NaOH until the colour changes from yellow to orange (~400μL).

Conditioned media was collected from BT-549 or MDA MB-231 cells that were transfected with isomiR mimics or stably expressing pre-miR-1307. Cells were incubated for two (transfected cells) or three days (stable cells) at 37°C and 5% CO_2_. Then, the media was changed to RPMI-1640 media containing only 2% FBS. After incubating for one additional day, media was collected and aliquoted. The aliquots were centrifuged at 3000rpm for 15min at 4°C. After transferring the supernatant to a new tube, the media was centrifuged at 4000rpm for 30min at 4°C and the aliquots were stored at -80°C.

For the sprouting assay, human umbilical vein endothelial cells (HUVECs) were cultured in Endopan 3 media containing 3% FBS and supplements at 37°C and 5% CO_2_ until they reached full confluency. Cells were harvested by trypsinization and transferred to a new tube. After centrifugation for 5min at 200 x g, the supernatant was removed and the cell pellet was resuspended in 3mL media. After counting, cells were distributed to 15mL falcon tubes with 20,000 cells per condition and media was added to a total volume of 7mL. Additional 1.75mL of the methocoel solution was added without creating bubbles. Using a multi-channel pipette, 25μl drops were placed onto a 10cm square petri dish, air bubbles were avoided. By inverting the plate, the now hanging drops were incubated at 37°C and 5% CO_2_ for 24h. Next, the drops were checked for spheroids using the microscope. Using 10mL PBS, the drops were gently washed off the plate and the spheroids were collected in 50mL tubes. After centrifugation at 200 x g for 5min, the supernatant was aspirated and the spheroids were resuspended in 3.5mL methocoel solution. Subsequently, 3.5mL of the collagen media was added (1:1) and carefully mixed by rolling the tubes in order to avoid air bubbles. Of this mix, 1mL for each condition was added per well in a 24-well low attachment plate and incubated for 30 min at 37°C and 5% CO_2_. After the collagen has polymerized, 200μL of respective conditioned media was added. As positive sprouting control, Endopan-3 media with FBS and supplements was added, as negative control Endopan-3 basal media (without supplements) was used. Plates with the now embedded spheroids were incubated for 24h at 37°C and 5% CO_2_. In order to stop the reaction, 1mL 10% paraformaldehyde per condition was added. Plates were then stored at 4°C for up to 4 weeks. Images of the spheroids were acquired with the Axiovert25 microscope with 5X magnification. Image analysis was performed using ImageJ and Microsoft Excel. The following parameters were analyzed: Number of sprouts and average length of sprouts. For the analysis, the average of ten spheroids per condition was taken.

*Secretome analysis by Mass Spectrometry*

For MS-based analysis of secreted proteins, 1*10^6^ MDA-MB-231 were seeded into 10 cm dishes with 10 mL full growth media (10% FBS). After 24h, the cells were transfected with miRNA mimics. Next, the medium was discarded and the cells were washed twice with 10 mL DPBS to thoroughly remove residual FBS from the media. The cells were incubated for 16 h in 10 mL RPMI without FBS. Supernatants were collected and transferred into a 15 mL tube on ice. Dead cells were removed from the conditioned media by centrifugation for 10 min at 4°C and 10,000 rpm (15,320 g). To concentrate the protein solution, it was transferred into a 3K MWCO spin column (Amicon) and centrifuged for 3 h at 4°C and maximum rpm. The protein concentrates were transferred into 2 mL reaction tubes and quantified using BCA staining.

Protein digestion was performed using the SP3 method [59]. Briefly, magnetic SP3 beads solution was prepared by mixing 20 µl beads with 20 µl beads B and adding 160 µl H_2_O. After incubation on a magnet for 1 min, the supernatant was removed and the beads were washed three times with 200 µl H_2_O and re-suspended to 20 µl in H_2_O.

For protein clean-up, 20 µg protein was diluted to a final volume of 60 µl in 100 mM TEAB buffer. CAA and TCEP were added to final concentrations of 40 mM and 10 mM, respectively. Reduction and alkylation of disulfide bridges was performed for 5 min at 95°C. Next, 2 µl beads preparation and 102 µl absolute ethanol were added by gently pipetting up and down. On a shaker at 650 rpm, protein precipitation and binding to the beads was performed for 15 min at room temperature. On the magnet, bound proteins were washed twice with 200 µl 80% ethanol and once with 200 µl 100% ACN. Finally, the bead-bound proteins were re-suspended in 75 µl 100 mM TEAB and sonicated for 30 s in the water bath. Trypsin was added at an enzyme-to-protein ratio of 1:25 and digestion was performed for 16 hours at 37°C. Digested peptides were vacuum-centrifuged to dryness.

For LC-MS/MS analysis, peptides were dissolved in ULC/MS grade water containing 0.1% trifluoracetic acid (TFA) and 2.5% 1,1,1,3,3,3-Hexafluoro-2-propanol (HFIP). The samples were transferred to autosampler vials and placed in the autosampler module of the Ultimate 3000 liquid chromatography system. The LC is operated at a flow of 300 nl/min and the columns were heated to 35°C. Peptides are loaded onto a trapping cartridge (Thermo Scientific, C18, 0.3 x 5 mm, Cat. No. 160454) in the presence of 98% loading buffer A (0.1% TFA in water) and 2% loading buffer B (0.1% TFA in acetonitrile). Peptides are eluted from the trapping column and loaded onto the analytical column (Waters, 186008795, BEH C18 130Å 1.7 µm 75x200 mm). Peptides were separated according to hydrophobicity with a linear gradient over the course of 102 min of 4-30% acetonitrile. In the mass spectrometer (Thermo, Orbitrap Exploris 480), peptides were analysed in DIA mode. MS1 scans are acquired at a resolution of 120K and cover the mass range of 350 - 1400 m/z. Maximum injection time is 45 ms and the automated gain control (AGC) target was set to 3e6 ions. MS2 scans were acquired for 47 precursor isolation windows of variable width for optimized window placement and 1 m/z overlap converting a mass range from 400 – 1000 m/z (NCE 28%, 30K resolution, maxIT 54 ms, AGC target 1e6 ions)Peptide and protein identification and quantification from DIA raw data was performed with the Biognosys software Spectronaut (version 15.5) in directDIA mode searching against the human proteome (downloaded from Uniprot on July 14^th^, 2020 ( 74,811 entries).

The mass spectrometry proteomics data together with a file containing the detailed search parameters (Spectronaut_search_parameters.txt) were deposited to the ProteomeXchange Consortium via the PRIDE partner repository [60] with the dataset identifier PXD041087. Protein quantitation data were log2 transformed (Supplementary Table 10) and further processed, i.e. they were filtered for potential secreted proteins according to the human protein atlas and filtered for those which were significantly changed for each miR in comparison to the combination of ctrl1 and 2. Results were z-scaled over all proteins and depicted in a heatmap using R (version 4.3) and the pheatmap package (version 1.0.12). Additionally, filtered data were used to generate a Venn diagram which shows the number of significantly changed proteins for each miRNA (p value < 0.05). R (version 4.3) and the eulerr package (version 7.0.0) were used for this purpose.

*COX-PH regression analysis*

To evaluate the prognostic value of miR-1307-3p and miR-1307-5p expressions in breast cancer, a multivariate COX Proportional Hazards (COX-PH) regression model was used. The model was generated based on the overall survival (OS) and end-point information of 933 breast cancer patients filtered as detailed above from TCGA BRCA dataset [61].

Due to the high correlation between the two 5’isomiRs derived from the 3’ arm of pre-miR-1307 (miR-1307-3p|0 and miR-1307-3p|1), their prognostic value could not be investigated separately. Therefore, RPM expressions of the two isomiRs were summed up yielding the total expression of the miR-1307-3p arm while the expression of the single 5’ isomiR (miR-1307-5p|0) was directly used as the expression of miR-1307-5p. log2-transformed rpm of the 3p- and the 5p-arm of pre-miR-1307 as well as the age of diagnosis for each patient were assigned as covariates while primary diagnosis and molecular subtype information of patients were used for stratification in the COX model.

Molecular subtype information which was used as a stratum included four categories: LumA, LumB, Her2, and Basal. The patients with Normal-like molecular subtype in the original TCGA BRCA confounder dataset had been excluded from the analyses as detailed above. The second stratum, primary diagnosis, originally contained seven categories in the TCGA BRCA confounder dataset. They were reduced to three in the COX model: The two predominant categories ‘infiltrating ductal carcinoma’ and ‘infiltrating lobular carcinoma’ were kept unchanged while the remaining five other types of primary diagnosis were converted to the categorical variable named as ‘others’. 95% confidence intervals (CI) for the hazard ratios were used and CI values, HRs and p-values for each estimate were displayed in a forest plot. All analyses were performed using survival (v.3.4.0) library and the forest plot was generated using forestmodel (v.0.6.2) library in R (v.4.1.3).

*Data analysis and visualization*

Analyses in R were performed using version 4.2.1 and R studio if not indicated differently. R packages used for analysis and visualization are summarized below. Some packages were used in many of the performed analyses and are not indicated separately with each step. For these packages, the version is given with the reference.

| R-package | See also |
| --- | --- |
| apeglm | Zhu A, Ibrahim JG, Love MI (2018). “Heavy-tailed prior distributions for sequence count data: removing the noise and preserving large differences.” Bioinformatics. doi:10.1093/bioinformatics/bty895. Version: 1.20.0 |
| BiomaRt | Mapping identifiers for the integration of genomic datasets with the R/Bioconductor package biomaRt. Steffen Durinck, Paul T. Spellman, Ewan Birney and Wolfgang  Huber, Nature Protocols 4, 1184-1191 (2009).  BioMart and Bioconductor: a powerful link between biological databases and microarray data analysis. Steffen Durinck, Yves Moreau, Arek Kasprzyk, Sean Davis,  Bart De Moor, Alvis Brazma and Wolfgang Huber, Bioinformatics 21, 3439-3440 (2005). |
| complexHeatmap | Gu Z, Eils R, Schlesner M (2016). “Complex heatmaps reveal patterns and correlations in multidimensional genomic data.” Bioinformatics. doi:10.1093/bioinformatics/btw313. |
| data.table | Dowle M, Srinivasan A (2022). _data.table: Extension of `data.frame`_. R package version 1.14.4, <https://CRAN.R-project.org/package=data.table>. Version: 1.14.4 |
| DESeq2 | Love MI, Huber W, Anders S (2014). “Moderated estimation of fold change and dispersion for RNA-seq data with DESeq2.” Genome Biology, 15, 550. doi:10.1186/s13059-014-0550-8. Version 1.38.0 |
| dplyr | Wickham H, François R, Henry L, Müller K, Vaughan D (2023). _dplyr: A Grammar of Data Manipulation_. R package version 1.1.2,  <https://CRAN.R-project.org/package=dplyr>. Version: 1.1.2 |
| edgeR | Robinson MD, McCarthy DJ and Smyth GK (2010). edgeR: a Bioconductor package for differential expression analysis of digital gene expression data. Bioinformatics  26, 139-140  McCarthy DJ, Chen Y and Smyth GK (2012). Differential expression analysis of multifactor RNA-Seq experiments with respect to biological variation. Nucleic Acids  Research 40, 4288-4297  Chen Y, Lun ATL, Smyth GK (2016). From reads to genes to pathways: differential expression analysis of RNA-Seq experiments using Rsubread and the edgeR  quasi-likelihood pipeline. F1000Research 5, 1438 |
| eulerr | Larsson J (2022). _eulerr: Area-Proportional Euler and Venn Diagrams with Ellipses_. R package, <https://CRAN.R-project.org/package=eulerr>. |
| forestmodel | https://github.com/NikNakk/forestmodel/ |
| GenomicFeatures | Lawrence M, Huber W, Pag\`es H, Aboyoun P, Carlson M, et al. (2013) Software for Computing and Annotating Genomic Ranges. PLoS Comput Biol 9(8): e1003118.  doi:10.1371/journal.pcbi.1003118 |
| GenomicRanges | Lawrence M, Huber W, Pag\`es H, Aboyoun P, Carlson M, et al. (2013) Software for Computing and Annotating Genomic Ranges. PLoS Comput Biol 9(8): e1003118.  doi:10.1371/journal.pcbi.1003118. Version: 1.49.0 |
| Ggplot2 | H. Wickham. ggplot2: Elegant Graphics for Data Analysis. Springer-Verlag New York, 2016. |
| Ggpubr | Kassambara A (2023). _ggpubr: 'ggplot2' Based Publication Ready Plots_. R package, <https://CRAN.R-project.org/package=ggpubr>. |
| Ggrepel | Slowikowski K (2023). ggrepel: Automatically Position Non-Overlapping Text Labels with 'ggplot2'. R package, https://github.com/slowkow/ggrepel. |
| matrixStats | Bengtsson H (2022). _matrixStats: Functions that Apply to Rows and Columns of Matrices (and to Vectors)_.  <https://CRAN.R-project.org/package=matrixStats>. Version 0.62.0 |
| Open xlsx | Schauberger P, Walker A (2022). _openxlsx: Read, Write and Edit xlsx Files_. R package, <https://CRAN.R-project.org/package=openxlsx>. |
| Pheatmap | Kolde R (2019). _pheatmap: Pretty Heatmaps_. R package, <https://CRAN.R-project.org/package=pheatmap>. |
| plyr | Hadley Wickham (2011). The Split-Apply-Combine Strategy for Data Analysis. Journal of Statistical Software, 40(1), 1-29. URL <https://www.jstatsoft.org/v40/i01/>. Version: 1.8.7 |
| RColorBrewer | Neuwirth E (2022). _RColorBrewer: ColorBrewer Palettes_, <https://CRAN.R-project.org/package=RColorBrewer>. Version 1.1-2 |
| RnBeads | Fabian Müller*, Michael Scherer*, Yassen Assenov*, Pavlo Lutsik*, Jörn Walter, Thomas Lengauer and Christoph Bock (2019) RnBeads 2.0: comprehensive analysis of DNA methylation data, Genome Biology, 20:55 |
| stringr | Wickham H (2022). _stringr: Simple, Consistent Wrappers for Common String Operations_. <https://CRAN.R-project.org/package=stringr>. Version: 1.5.0 |
| SummarizedExperiment | Morgan M, Obenchain V, Hester J, Pagès H (2022). _SummarizedExperiment: SummarizedExperiment container_.  <https://bioconductor.org/packages/SummarizedExperiment>. Version 1.28.0 |
| Survival | Therneau T (2023). A Package for Survival Analysis in R. R package, https://CRAN.R-project.org/package=survival.  Terry M. Therneau, Patricia M. Grambsch (2000). Modeling Survival Data: Extending the Cox Model. Springer, New York. ISBN 0-387-98784-3. |
| TCGAbiolinks | Colaprico A, Silva TC, Olsen C, Garofano L, Cava C, Garolini D, Sabedot T, Malta TM, Pagnotta SM, Castiglioni I, Ceccarelli M, Bontempi G, Noushmehr H (2015). “ TCGAbiolinks: An R/Bioconductor package for integrative analysis of TCGA data.” Nucleic Acids Research. doi:10.1093/nar/gkv1507, http://doi.org/10.1093/nar/gkv1507.  Silva, C T, Colaprico, Antonio, Olsen, Catharina, D'Angelo, Fulvio, Bontempi, Gianluca, Ceccarelli, Michele, Noushmehr, Houtan (2016). “TCGA Workflow: Analyze cancer genomics and epigenomics data using Bioconductor packages.” F1000Research, 5.  Mounir, Mohamed, Lucchetta, Marta, Silva, C T, Olsen, Catharina, Bontempi, Gianluca, Chen, Xi, Noushmehr, Houtan, Colaprico, Antonio, Papaleo, Elena (2019). “New functionalities in the TCGAbiolinks package for the study and integration of cancer data from GDC and GTEx.” PLoS computational biology, 15(3), e1006701. |
| tibble | Müller K, Wickham H (2023). _tibble: Simple Data Frames_. <https://CRAN.R-project.org/package=tibble>. Version: 3.1.7 |
| tidyr | Wickham H, Vaughan D, Girlich M (2023). _tidyr: Tidy Messy Data_. <https://CRAN.R-project.org/package=tidyr>. |
| Tidyverse | Wickham H, Averick M, Bryan J, Chang W, McGowan LD, François R, Grolemund G, Hayes A, Henry L, Hester J, Kuhn M, Pedersen TL, Miller E, Bache SM, Müller K, Ooms  J, Robinson D, Seidel DP, Spinu V, Takahashi K, Vaughan D, Wilke C, Woo K, Yutani H (2019). “Welcome to the tidyverse.” _Journal of Open Source Software_,  *4*(43), 1686. doi:10.21105/joss.01686 <https://doi.org/10.21105/joss.01686>. |

**References**

18. Li, X., et al., *5'isomiR-183-5p|+2 elicits tumor suppressor activity in a negative feedback loop with E2F1.* J Exp Clin Cancer Res, 2022. **41**(1): p. 190.

38. Liberzon, A., et al., *The Molecular Signatures Database (MSigDB) hallmark gene set collection.* Cell Syst, 2015. **1**(6): p. 417-425.

39. Fellmann, C., et al., *An optimized microRNA backbone for effective single-copy RNAi.* Cell Rep, 2013. **5**(6): p. 1704-13.

40. Soneoka, Y., et al., *A transient three-plasmid expression system for the production of high titer retroviral vectors.* Nucleic Acids Res, 1995. **23**(4): p. 628-33.

41. Langmead, B., et al., *Ultrafast and memory-efficient alignment of short DNA sequences to the human genome.* Genome Biol, 2009. **10**(3): p. R25.

42. Bonfield, J.K., et al., *HTSlib: C library for reading/writing high-throughput sequencing data.* Gigascience, 2021. **10**(2).

43. Danecek, P., et al., *Twelve years of SAMtools and BCFtools.* Gigascience, 2021. **10**(2).

44. Quinlan, A.R., *BEDTools: The Swiss-Army Tool for Genome Feature Analysis.* Curr Protoc Bioinformatics, 2014. **47**: p. 11 12 1-34.

45. Quinlan, A.R. and I.M. Hall, *BEDTools: a flexible suite of utilities for comparing genomic features.* Bioinformatics, 2010. **26**(6): p. 841-2.

46. Ibing, S., et al., *On the impact of batch effect correction in TCGA isomiR expression data.* NAR Cancer, 2021. **3**(1): p. zcab007.

47. Funakoshi, K., et al., *Highly sensitive and specific Alu-based quantification of human cells among rodent cells.* Sci Rep, 2017. **7**(1): p. 13202.

48. Holm, K., et al., *An integrated genomics analysis of epigenetic subtypes in human breast tumors links DNA methylation patterns to chromatin states in normal mammary cells.* Breast Cancer Res, 2016. **18**(1): p. 27.

49. Iorio, F., et al., *A Landscape of Pharmacogenomic Interactions in Cancer.* Cell, 2016. **166**(3): p. 740-754.

50. Zhou, W., et al., *SeSAMe: reducing artifactual detection of DNA methylation by Infinium BeadChips in genomic deletions.* Nucleic Acids Res, 2018. **46**(20): p. e123.

51. Assenov, Y., et al., *Comprehensive analysis of DNA methylation data with RnBeads.* Nat Methods, 2014. **11**(11): p. 1138-1140.

52. Muller, F., et al., *RnBeads 2.0: comprehensive analysis of DNA methylation data.* Genome Biol, 2019. **20**(1): p. 55.

53. Chen, Y.A., et al., *Discovery of cross-reactive probes and polymorphic CpGs in the Illumina Infinium HumanMethylation450 microarray.* Epigenetics, 2013. **8**(2): p. 203-9.

54. Houseman, E.A., et al., *DNA methylation arrays as surrogate measures of cell mixture distribution.* BMC Bioinformatics, 2012. **13**: p. 86.

55. Sturm, G., et al., *Comprehensive evaluation of transcriptome-based cell-type quantification methods for immuno-oncology.* Bioinformatics, 2019. **35**(14): p. i436-i445.

56. Becht, E., et al., *Estimating the population abundance of tissue-infiltrating immune and stromal cell populations using gene expression.* Genome Biol, 2016. **17**(1): p. 218.

57. Tetzlaff, F., et al., *MPDZ promotes DLL4-induced Notch signaling during angiogenesis.* Elife, 2018. **7**.

58. Tetzlaff, F. and A. Fischer, *Human Endothelial Cell Spheroid-based Sprouting Angiogenesis Assay in Collagen.* Bio Protoc, 2018. **8**(17): p. e2995.

59. Hughes, C.S., et al., *Ultrasensitive proteome analysis using paramagnetic bead technology.* Mol Syst Biol, 2014. **10**(10): p. 757.

60. Perez-Riverol, Y., et al., *The PRIDE database resources in 2022: a hub for mass spectrometry-based proteomics evidences.* Nucleic Acids Res, 2022. **50**(D1): p. D543-D552.

61. Berger, A.C., et al., *A Comprehensive Pan-Cancer Molecular Study of Gynecologic and Breast Cancers.* Cancer Cell, 2018. **33**(4): p. 690-705 e9.
